# Testing the efficiency of capture methods for questing *Hyalomma lusitanicum* (Acari: Ixodidae), a vector of Crimean-Congo hemorrhagic fever virus

**DOI:** 10.1093/jme/tjad127

**Published:** 2023-09-13

**Authors:** Raúl Cuadrado-Matías, Laia Casades-Martí, Alfonso Peralbo-Moreno, Sara Baz-Flores, Edgar García-Manzanilla, Francisco Ruiz-Fons

**Affiliations:** Health and Biotechnology (SaBio) Group, Instituto de Investigación en Recursos Cinegéticos (IREC), CSIC-UCLM-JCCM, Ronda de Toledo 12, 13005 Ciudad Real, Spain; Health and Biotechnology (SaBio) Group, Instituto de Investigación en Recursos Cinegéticos (IREC), CSIC-UCLM-JCCM, Ronda de Toledo 12, 13005 Ciudad Real, Spain; Health and Biotechnology (SaBio) Group, Instituto de Investigación en Recursos Cinegéticos (IREC), CSIC-UCLM-JCCM, Ronda de Toledo 12, 13005 Ciudad Real, Spain; Health and Biotechnology (SaBio) Group, Instituto de Investigación en Recursos Cinegéticos (IREC), CSIC-UCLM-JCCM, Ronda de Toledo 12, 13005 Ciudad Real, Spain; Pig Development Department, Teagasc Grassland Research and Innovation Centre, Moorepark, Fermoy, County Cork P61 C996, Ireland; School of Veterinary Medicine, University College Dublin, Belfield, Dublin 4, Ireland; Health and Biotechnology (SaBio) Group, Instituto de Investigación en Recursos Cinegéticos (IREC), CSIC-UCLM-JCCM, Ronda de Toledo 12, 13005 Ciudad Real, Spain; CIBERINFEC-CIBER de Enfermedades Infecciosas, Centro de Investigación Biomédica en Red de Enfermedades Infecciosas, Instituto de Salud Carlos III, Av. Monforte de Lemos, 3-5. Pabellón 11. Planta 0, 28029 Madrid, Spain

**Keywords:** density, *Hyalomma* spp, method testing, population size, questing tick

## Abstract

Available methods to census exophilic tick populations have limitations in estimating true population size due to their inability to capture a high proportion of the actual tick population. We currently ignore the efficacy of these methods to capture questing *Hyalomma* spp. ticks, vectors of the Crimean-Congo hemorrhagic fever virus. To address the need of accurately estimating questing densities of *Hyalomma* spp., we designed a field experiment to test the efficacy of blanket dragging, blanket flagging, CO_2_-baited traps, and an ad hoc designed method, absolute surface counts, in capturing adult *Hyalomma lusitanicum* ticks from known numbers of preset fluorescent-marked ticks. The experiment was designed in 2 stages to estimate the point (1-day sampling) and cumulative (3-day serial sampling) efficacy of the methods under varying sampling effort and habitat. Tick survival, host interference, and weather effects on efficacy were controlled for in multiple regression models. There was high variability in method efficacy for capturing ticks, which was also modulated by effort, habitat, tick density, hosts, and soil temperature. The most effective method was absolute surface counts for both point estimates (39%) and cumulative efficacy (83%). CO_2_-baited traps reached a maximum efficacy of 37%, while blanket dragging and blanket flagging captured a maximum of the 8% of the marked ticks. Our results reveal the strengths and weaknesses of the different tick capture methods applied to adult *H. lusitanicum* and lay the groundwork for more accurate inferences about the true size of exophilic tick populations.

## Graphical Abstract

**Graphical Abstract:**
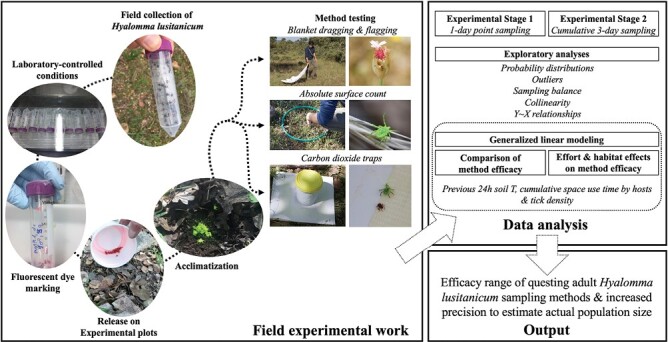

## Introduction

The relevance of ticks from a health perspective derives from the deleterious effects that these hematophagus parasites have on their hosts. As obligate blood-feeding arthropods, ticks draw blood from vertebrate hosts. This has negative consequences on the physiology of the hosts, and even compromises their survival, e.g., parasitization by the winter tick (*Dermacentor albipictus*) of North American wild ungulates (Calvente et al. 2020). Undoubtedly, the greatest negative health impact of tick parasitism is the transmission of pathogens that can compromise the health and survival of their hosts (Madison-Antenucci et al. 2020).

An essential parameter in the ecology of tick-borne diseases, namely tick population size, has received little attention in epidemiological research (Daniels et al. 2018, Jaenson et al. 2018). However, without the estimation of basic tick demographic parameters, it is difficult to draw accurate conclusions about the ecology and dynamics of tick-borne pathogens and, therefore, promote cost-effective prevention and/or control strategies. A wide variety of monitoring techniques exist to estimate tick population size, the choice of which depends on the behavioral ecology of the tick species. Exophilic (non-nidicolous) ticks are those that actively seek hosts from their harborage sites in the environment, e.g., under vegetation or within fragmented rocky soils (Sobrino et al. 2012). While some exophilic ticks search for hosts using an ambush strategy, e.g., ticks of the *Ixodes ricinus* complex, others such as several *Hyalomma* spp. and *Amblyomma* spp. ticks additionally exhibit an active host-hunting strategy. Hunting ticks may also behave as ambush ticks when environmental conditions are favorable for host searching (Ramos et al. 2017).

One of the most commonly used exophilic tick population tracking techniques uses a cotton or wool fuzzy cloth of known surface area to search for questing ticks on vegetation tips (Salomon et al. 2020). This technique takes advantage of the ambush host-seeking behavior of many exophilic tick species by simulating the passage of a host to which questing ticks can attach. By checking the cloth periodically, usually every 5, 10, or 15 m of dragged surface (Nyrhilä et al. 2020, Salomon et al. 2020), specimens that are active in the vegetation can be counted and their abundance estimated (Kjellander et al. 2021). Dragging or flagging collects only a proportion of the tick population that is actively questing on the vegetation (Daniels et al. 2018). In drag sampling, the sampler pulls the cloth dragging it behind the vegetation surface, while in flagging the observer more gently passes the cloth over the vegetation surface lateral to the line of progression on the transect (see details in Salomon et al. 2020). Nyrhilä et al. (2020) estimated that blanket dragging picks up only 6 %, on average, of the *I. ricinus* tick population. Similarly, Tälleklint-Eisen and Lane (2000) reported a 5.9% efficiency of simple drag sampling on *Ixodes pacificus* nymphs. Dragging or flagging a cotton cloth on vegetation does not make a difference in the proportion of the tick population sampled (Rulison et al. 2013).

Other tick sampling methods take advantage of tick chemotaxis to carbon dioxide exhaled by vertebrate hosts by setting CO_2_-releasing traps to attract and collect them (Caiado et al. 1990). However, many exophilic ticks show variations in their attraction to carbon dioxide and this impairs the effectiveness of the method. Kensinger and Allan (2011) found that CO_2_-baited traps were capable of collecting about 16% of previously marked adult *Amblyomma americanum* ticks. Different versions of flagging, dragging, and CO_2_-trapping methods have been evaluated on some tick species with varying efficacies (Mays et al. 2016). This complicates the choice of a method for estimating exophilic tick density, especially if the efficacy of the methods has never been tested before for the target tick species.

The genus *Hyalomma* includes 27 species distributed throughout Asia, Africa, and Europe (Guglielmone et al. 2014). They are vectors of important animal pathogens and Crimean-Congo hemorrhagic fever virus (CCHFV) for humans (Bente et al. 2013). However, CCHF did not emerge outside economically depressed areas of the world until the beginning of the 21st century (Filiâtre et al. 2019) and little attention has been paid to the ecology of *Hyalomma* spp. ticks in the scientific literature. The efficacy of the different potential methods to estimate the population size of *Hyalomma* spp. is unknown. Consequently, we cannot estimate the real size of the *Hyalomma* spp. population infected by a particular infectious agent and, therefore, the real risk of pathogen transmission to vertebrate hosts. In the Iberian Peninsula, the most abundant species of the genus *Hyalomma* is *Hyalomma lusitanicum* (Ruiz-Fons et al. 2006, 2013), which, together with *Hyalomma marginatum*, is responsible for the maintenance and transmission of CCHFV (Spengler and Bente 2017) and for the recent cases of CCHF reported in Spain (Negredo et al. 2017, Sánchez-Seco et al. 2021). Only adult stages of *H. lusitanicum* are considered relevant vectors of CCHFV due to the low frequency of bites of immature stages of *Hyalomma* spp. in humans. (Merino et al. 2005, Vieira Lista et al. 2022). Both *H. lusitanicum* and *H. marginatum* show important differences in ecology and behavior (Valcárcel et al. 2020). Adult stages of *H. lusitanicum* and *H. marginatum* are highly specialized in parasitizing large animals, mainly ungulates (Ruiz-Fons et al. 2013). *Hyalomma lusitanicum* is abundant in wild environments with high densities of wild ungulates while *H. marginatum* is associated with domestic livestock, mainly cattle and horses (Valcárcel et al. 2020). Both species can move rapidly towards passing hosts, so they are considered host-hunting ticks. However, they can also be found ambushing hosts in vegetation. It is unknown how these species select whether to hunt or ambush a host, so population monitoring methods should take this plastic host-seeking behavior into account.

The increasing public health importance of *Hyalomma* spp. ticks in Europe, Africa, and the Middle East requires filling existing gaps in the accuracy of census tools for estimating population size. This study aims to estimate the efficacy of commonly used and novel tick capture methods for inferring accurate estimates of *H. lusitanicum* tick population size and to identify which are the main determinants of variation in efficacy. This would allow the selection of the most efficient method or a set of complementary methods to accurately estimate *H. lusitanicum* population size.

## Materials and Methods

### Study Area

The experiment was conducted entirely on a 6,000 ha publicly owned land (QM) located in west-central Spain (latitude: 39.397709°; longitude: −4.076207°). The habitat is dominated by large areas of grasslands mixed with scattered shrubs, trees covering the valley bottoms, and patches of Mediterranean forest/shrubs on the mountain slopes. Red deer (*Cervus elaphus*) and Eurasian wild boar (*Sus scrofa*) are the predominant ungulates in QM and both are very abundant (Casades-Martí et al. 2020), resulting in high tick densities. *Hyalomma lusitanicum* stands out as the most abundant tick species in QM. Roe deer (*Capreolus capreolus*) are also present in QM at low densities (Martinez-Guijosa et al. 2020) and harbor adult *H. lusitanicum* ticks as well.

### Selection of Tick Capture Methods

For the experiment, we selected 3 methods commonly used for capturing questing ticks (Cohnstaedt et al. 2012, Mays et al. 2016): (i) blanket—cotton cloth—dragging (BD); (ii) blanket—cotton cloth—flagging (BF); and (iii) CO_2_ trapping (CDT). The cotton fabrics used were white 1 × 1 m pieces of terry toweling attached at one end to 1.5 m wooden poles. A 3-m rope was attached to both ends of the stick to drag the blanket. Carbon dioxide-based traps consisted of 1-liter, thick-walled (5 mm) cylindrical plastic containers topped with a lid. Two centimeters from the base of each container, we drilled 8 evenly distributed holes of 0.5 cm diameter. During field sampling, the containers were placed on white PVC sheets of 5 mm thick and 45 × 45 cm provided with double-sided adhesive tape (TESA, Hamburg, Germany) on each of their 4 sides. The traps were baited with 500 g of 16-mm dry ice pellets. A 150-ml cold accumulator at −80 °C per trap was included to slow the rate at which the dry ice sublimated. The time of placement and collection of each trap, which was active for 3 h, was recorded. All CO_2_-capture trials were conducted between 8:30 AM and 12:30 PM. In addition, based on the hunting behavior of *H. lusitanicum* ticks, we designed a method focused on the activation and collection of the ticks present in an area of 0.16 m^2^ delimited with a plastic ring of 45 cm diameter. This method was termed absolute surface count (ASC) because it is expected to detect most of the ticks enclosed by the hoop (except for inactive ticks hiding under the ground or the leaflitter). The ASC consisted of randomly placing the hoop on the soil surface, gently breathing over the area enclosed by the hoop for 15 s, and collecting the observed ticks. Any tick that, being outside the hooped area, moved into the hoop during sampling were specifically discarded.

For each sampling method, we wanted to estimate whether an increase in sampling effort would improve the efficacy of the method for adult *H. lusitanicum* ticks. Therefore, we designed the experiment with an increasing effort gradient of 3 classes for each method: low, medium, and high. For CO_2_ traps we selected only low and medium efforts due to the expected attraction overlap between traps in a small experimental plot (Kensinger and Allan 2011). For blanket dragging/flagging, the low, medium, and high efforts were 1, 5, and 10 m per plot, respectively. Low and medium efforts for CO_2_ capture were 1 or 2 traps, respectively, per study plot. For the ASC method, the low, medium, and high efforts consisted of 4, 8, or 12 replicates, respectively, per plot.

### Experiment Design and Implementation

In April 2020, more than 3,500 adult specimens of *H. lusitanicum* were captured in QM by flagging the vegetation with 1 m^2^ cotton cloths to collect local wild-behaved specimens at enough numbers for use in the experiment. Ticks were morphologically identified with specific taxonomic keys (Estrada-Peña et al. 2018), transferred to containers covered with appropriately sized mesh netting in batches of 200 individuals, and assigned to chambers maintaining a temperature of 20 ± 2 °C and relative air humidity of 85 ± 5% under a 12:12 h light–dark regime. Conditions in the chambers resembled those of spring in south-central Spain. Ticks were kept in these conditions until they were selected for the experiment. The experiment was designed in 2 stages.

#### First stage of the experiment

**Fig. 1.**
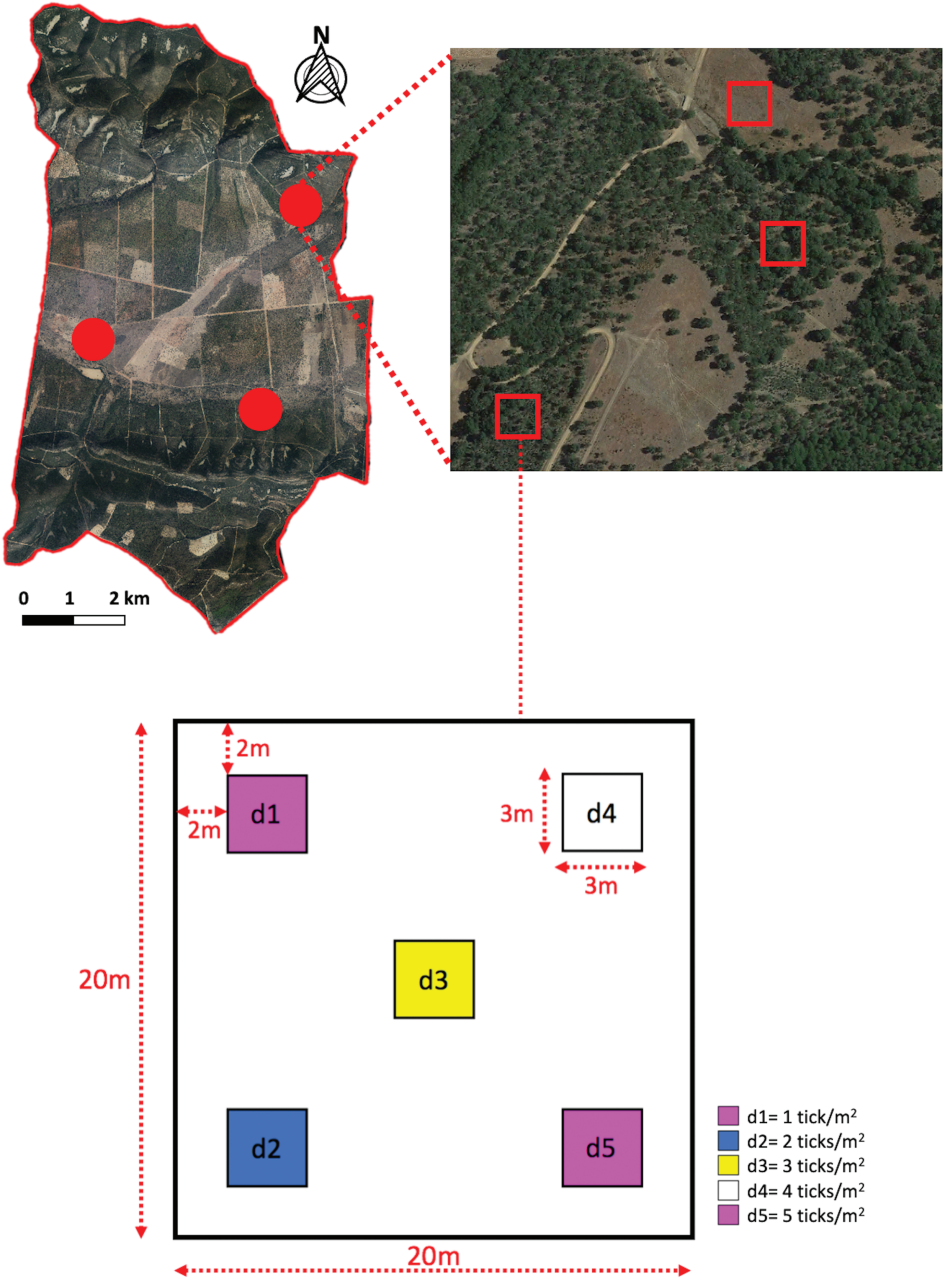
Location of study replicates and plots within the boundaries of the study land (south-central Spain). Circles in the upper left image represent each of the 3 replicates in the study area, and squares in the upper right image indicate each of the 3 selected 20 × 20 m habitat plots per replicate. The schematic representation of a 20 × 20 m study plot and the allocation of different experimental tick densities to 3 × 3 m plots within it are displayed at the bottom of the figure. Ticks in density plots d1 and d5 were marked with pink fluorescent dye. Ticks in density plot d2 were marked with blue fluorescent dye, those in plot d3 with yellow fluorescent dye and those in plot d4 with white fluorescent dye.

In the first stage of the experiment, we sought to estimate the point efficiency (single sampling) of methods with a gradient of increasing sampling effort for the 3 main habitats in which *H. lusitanicum* lives in Mediterranean environments: (i) grassland; (ii) shrubland; and (iii) forest (Valcárcel et al. 2020). We selected different habitats because grassland areas are highly exposed to solar radiation, while shrublands and forests present an increasing gradient of vegetation cover that buffers soil moisture and modulates the hydric stress experienced by ticks (Ruiz-Fons and Gilbert 2010). The experiment was conducted in 3 replicates per habitat. In each replicate, a 20 × 20 m plot containing five 3 × 3 m plots was delimited (Fig. 1). Each 3 × 3 m plot was assigned one of the 5 *H. lusitanicum* adult densities selected according to previous estimates (the authors, unpublished), from 1 to 5 ticks/m^2^ (sex ratio: 1:1). The ticks were marked with 4 different colors of innocuous fluorescent dyes (pink, green, blue, and white; DayGlo Color Corp., Cleveland, USA). Ticks at each density were marked with a different color, except those in the 1 and 5 ticks/m^2^ plots, which were marked with pink dye. The 3 × 3 m plots with 1 and 5 ticks/m^2^ were assigned in opposite corners of each 20 × 20 m plot to avoid interference from possible tick movement between plots.

Ticks were released on 4 May 2020 in the center of each 3 × 3 m plot and allowed a 2-day acclimation period. Sampling was initiated on 6 May 2020 (Table 1). All 4 tick capture methods were run simultaneously at the different tick densities per replicate, so that at least 3 samples per method and effort were taken on consecutive days (Table 1). With this approach, we achieved that each method and effort had at least 3 simultaneous replicates per tick density, habitat, and sampling day. Captured ticks were returned to their plots immediately after sampling. Potential tick movements out of each 3 × 3 m plot were monitored daily for the duration of the experiment. Monitoring was carried out immediately before sampling every day by dragging a blanket over a 2-m band surrounding each 3 × 3 m plot, and actively breathing along this 2-m band in a direction opposite to that of the experimental plot. Any marked ticks detected were returned to their plots. In no case, did we find a marked tick from a particular plot in the vicinity of its nearest plots. This stage of the experiment was conducted within the main period of annual activity of adult *H. lusitanicum* in continental Spain (April–June; Valcárcel et al. 2020).

**Table 1.** Study schedule and design for experimental stage 1

Hab^a^				Low effort^c^					Medium effort^d^						High effort^e^		
	Td^b^	Day 1	Day 2	Day 3	Day 4	Day 5	Day 6	Day 7	Day 8	Day 9	Day 10	Day 11	Day 12	Day 13	Day 14	Day 15	Day 16
Grassland 1	d1–d5	Acclimatization		**A**	**B**	**C**	Acclimatization		**F**	**D**	**G**	**E**	Acclimatization		**I**	**J**	**H**
Grassland 2	d1–d5			**B**	**C**	**A**			**D**	**G**	**E**	**F**			**J**	**H**	**I**
Grassland 3	d1–d5			**C**	**A**	**B**			**G**	**E**	**F**	**D**			**H**	**I**	**J**
Shrubland 1	d1–d5			**B**	**C**	**A**			**D**	**F**	**G**	**E**			**J**	**H**	**I**
Shrubland 2	d1–d5			**C**	**A**	**B**			**G**	**E**	**F**	**D**			**H**	**I**	**J**
Shrubland 3	d1–d5			**A**	**B**	**C**			**F**	**D**	**G**	**E**			**I**	**J**	**H**
Forest 1	d1–d5			**C**	**A**	**B**			**G**	**E**	**F**	**D**			**H**	**I**	**J**
Forest 2	d1–d5			**A**	**B**	**C**			**F**	**D**	**G**	**E**			**I**	**J**	**H**
Forest 3	d1–d5			**B**	**C**	**A**			**D**	**G**	**E**	**F**			**J**	**H**	**I**

^a^Habitat type of the 20 × 20 m plots (numbers indicate habitat replicates).

^b^Tick density: **d1** = 1/m^2^, **d2** = 2/m^2^, **d3** = 3/m^2^, **d4** = 4/m^2^, **d5** = 5/m^2^.

^c^ **A** = 1 m drag-flag/plot, **B** = 4 absolute surface counts (ASC)/plot, **C** = 1 CO_2_ trap/plot.

^d^ **D** = 5 m drag/plot, **E** = 2 CO_2_ traps/plot, **F** = 5 m flag/plot, **G** = 8 ASC/plot.

^e^ **H** = 10 m drag/plot, **I** = 12 ASC/plot, **J** = 10 m flag/plot.

#### Second stage of the experiment

In the second stage, we sought to estimate the cumulative effectiveness of the methods in continuous serial sampling trials, also with a gradient of increasing sampling effort and in the same habitats. The location of the 20 × 20 m plots and the 3 × 3 m plots within them was slightly modified in the second stage of the experiment from that of the first stage to avoid interference. The ticks released in the second stage of the experiment were different from those in the first experimental stage but came from those collected in the study land in April. In this second one, we only analyzed 2, 3, and 4 ticks/m^2^, and only considered one CO_2_ trap effort per plot.

On 29 May 2020, we released the marked ticks into their plots and allowed them to acclimate for 2 days (Table 2). Sampling was repeated on 3 consecutive days per replicate, method, and effort. Unlike what we did in the first stage, ticks collected on days 1 and 2 of each 3-day series in each plot were kept in properly labeled 15-ml tick transport containers and maintained under laboratory-controlled environmental conditions until day 3. After the third day of sampling, all ticks collected in the 3-day series were returned to their plot and allowed 1 day of acclimatization between samplings (see Table 2). As in the first stage, ticks that moved out of the 3 × 3 m plots were returned to their home plot. This stage of the experiment was also conducted within the annual activity period of adult *H. lusitanicum* (Valcárcel et al. 2020).

**Table 2.** Study schedule and design for experiment 2

				Low effort^c^				Medium effort^d^				High effort^d^		
Hab^a^	Td^b^	Day 1	Day 2	Day 3	Day 4	Day 5	Day 6	Day 7	Day 8	Day 9	Day 10	Day 11	Day 12	Day 13
Grassland 1	**d2–d4**	Acclimatization		**A**	**A**	**A**	Acclimatization	**E**	**E**	**E**	Acclimatization	**I**	**I**	**I**
Grassland 2	**d2–d4**			**C**	**C**	**C**		**D**	**D**	**D**		**H**	**H**	**H**
Grassland 3	**d2–d4**			**B**	**B**	**B**		**F**	**F**	**F**		**G**	**G**	**G**
Shrubland 1	**d2–d4**			**C**	**C**	**C**		**D**	**D**	**D**		**H**	**H**	**H**
Shrubland 2	**d2–d4**			**B**	**B**	**B**		**F**	**F**	**F**		**G**	**G**	**G**
Shrubland 3	**d2–d4**			**A**	**A**	**A**		**E**	**E**	**E**		**I**	**I**	**I**
Forest 1	**d2–d4**			**B**	**B**	**B**		**F**	**F**	**F**		**G**	**G**	**G**
Forest 2	**d2–d4**			**A**	**A**	**A**		**E**	**E**	**E**		**I**	**I**	**I**
Forest 3	**d2–d4**			**C**	**C**	**C**		**D**	**D**	**D**		**H**	**H**	**H**

^a^Habitat type of the 20 × 20 m plots (numbers indicate habitat replicates).

^b^Tick density: **d2** = 2/m^2^, **d3** = 3/m^2^, **d4** = 4/m^2^.

^c^ **A** = 1 m drag-flag/plot, **B** = 4 absolute surface counts (ASC)/plot, **C** = 1 CO_2_ trap/plot.

^d^ **D** = 5 m drag/plot. **E** =8 ASC/plot, **F** = 5 m flag/plot.

^e^ **G** = 10 m drag/plot, **H** = 12 ASC/plot, **I** = 10 m flag/plot.

### Statistical Analysis

#### Estimating factors that may interfere with capture efficiency

The choice of a field experimental trial carries potential limitations due to the presence of tick hosts that could mop off ticks from the plots throughout the experiment (Daniels et al. 2018). To control for host interference, we set 2 camera traps (Trophy Cam HD Aggressor No-Glow Trail Camera, Bushnell, USA) per replicate that were in operation from the day before tick release until the end of each stage of the experiment. The camera traps were fixed 40 cm above the ground to metal bars arranged at 2 corners of the 20 × 20 m plot, focusing on the interior of the plot. They were programmed to take images continuously with each activation with an interval of one second between images. Their operation was monitored with automatic images taken at 3 AM and 3 PM each day. Camera traps were checked every 2–3 days. The sensitivity of the cameras was adjusted to moderate values to detect the presence of potential *H. lusitanicum* adult hosts (ungulates) at a maximum distance of 10 m (Palencia et al. 2022). The images captured by the 2 camera traps per plot were reviewed to extract the images in which animals were recorded. Only those ungulates captured by camera traps at a distance ≤10 m were considered (Palencia et al. 2022). We assumed that the probability of ticks attaching to a host is a linear function of the time hosts remain in the space where ticks have been released (Hofmeester et al. 2017). Therefore, we estimated the cumulative time of plot space use by hosts from tick release to the sampling start time (recorded specifically for each replicate plot and sampling day) at each stage of the experiment. We divided wild ungulate records into visits. A visit was defined as the time of continuous recording of one or more individuals of the same species (there were no simultaneous records of different species) by a camera trap. Two contiguous images recorded with a time difference of 10 s were considered to belong to independent host visits. The duration of each visit was estimated and this duration was multiplied by the number of individuals recorded during the visit to calculate a time value per individual host. The cumulative time of visits per host between tick release and each sampling was estimated independently per camera trap, but the final index was the result of averaging the recording times of the 2 cameras placed per 20 × 20 m plot. The values of the estimated indices for the nine replicates and the 2 phases of the experiment are shown in Supplementary Figs. S1 and S2.

Natural mortality of ticks could also have an effect on the number of experimental ticks available in the study plots to be captured (Daniels et al. 2018). To estimate the natural mortality of adult *H. lusitanicum* ticks during the experiment, we used round plastic containers 12 cm in diameter and 8 cm high covered with a glued mesh of 1-mm pore diameter at the top and bottom. The containers were placed semiburied at a depth of 5 cm under the soil of each plot to allow moisture exchange between the soil and the container; the top was covered with local organic material. Containers were stocked with 20 adult *H. lusitanicum* (sex ratio 1:1), 10 marked with fluorescent dyes and 10 unmarked. Two containers per study replicate were placed on the day ticks were released in the study plots until the end of each experiment. The containers were checked each sampling day to record tick status (live/dead) and number of ticks. Records of the number of dead ticks in the containers were used to calculate the mortality rate per study plot. The mortality rate was estimated as the proportion of ticks dying throughout the experimental stage relative to the initial number of ticks per 20x20 m sampling plot.

Finally, environmental abiotic conditions could also influence tick questing activity and capture efficiency estimation. Therefore, we measured soil temperature and moisture variations with a 10-min precision throughout the experiment by placing a data logger (Tinytag TGP-4500, Gemini Data Loggers, UK) per 20 × 20 m plot that was active throughout the study (see Supplementary Fig. S3). We extracted the soil temperature and moisture values recorded per plot for the exact time at which each sampling was conducted. In addition, we estimated the mean soil temperature and moisture for the 24 h prior to each sampling with the 10-min records from the data loggers as a measure of the influence of recent abiotic conditions on tick activity. The coefficient of variation of the 10-min temperature and moisture measurements taken in the 24 h prior to each sampling was estimated as a measure of the variation in abiotic environmental conditions to which ticks were exposed in the soil. This was done because high variations in soil temperature and humidity could result in lower tick activity, whereas more stable values could determine more favorable conditions for tick host search.

#### Estimation of the capture efficiency of the methods

We initially performed an exhaustive exploratory analysis of the data (Zuur et al. 2010). We analyzed the presence of possible sampling imbalances and the associative relationships between explanatory covariates (Pearson correlation matrix) to avoid multicollinearity effects. In addition, we analyzed the univariate associations of the explanatory variables (method, habitat, effort, host space use, soil temperature and moisture at the time of sampling and 24 h before sampling, and variation in soil temperature and moisture 24 h before sampling) with the response variable (probability of capture of marked ticks) as a means of choosing between sets of highly correlated explanatory variables. Next, the probability of capture of released ticks per 3 × 3 m plot was modeled as a function of several explanatory variables using generalized linear models. Continuous predictors were mathematically transformed using logarithms (log_10_(*x*)+1) to homogenize the diversity of scales at which the different parameters were measured. We modeled the probability of tick capture separately for the first and second stages of the experiment.

We initially analyzed the existence of relevant differences in point capture efficiency among the 4 methods in the specific context of *H. lusitanicum* density in the plots, the cumulative time of host use of the plot space before sampling and the mean value of soil temperature in the 24 h prior to sampling. Both selected continuous predictors showed the strongest effects on capture efficiency in the descriptive analysis with respect to other possible modulating parameters (i.e., soil temperature and moisture at the time of sampling, soil moisture in the 24 h prior to sampling, and variation in soil temperature and moisture in the 24 h prior to sampling). After observing differences between methods, we separately modeled the capture probability of each method as a function of effort, habitat, tick density, host space usage, and soil temperature in the previous 24 h. The number of ticks captured over the total number of ticks released in each plot was the response variable of the binary model. Generalized linear models were constructed with a binomial probability distribution and a logit link function using the “glm” command of the R statistical package “stats.”

The cumulative captures of marked *H. lusitanicum* obtained on day 3 of each 3-day serial sampling of the second stage of the experiment were modeled to estimate whether capture efficiency varied among methods, efforts, and habitats with sampling repetitions on consecutive days. To do this, the number of ticks captured on the 3 sampling days was summed. Models were constructed as described for stage 1 of the experiment.

For each of the selected models, we estimated the probability of capturing adult *H. lusitanicum* as a function of different levels of the categorical predictors (method, effort, habitat, and tick density) to explore differences between factor levels. This was done by calculating the estimated marginal means for these parameters using the “lsmeans” function of the “emmeans” package of R. We additionally estimated the capture odds for each paired level of the categorical variables included in any of the models to compare in a paired fashion the capture efficiency between different levels of these predictors.

## Results

### First Stage of the Experiment

The results of the exploratory Pearson correlation analysis between the continuous predictors considered for modeling are shown in Supplementary Fig. S4. The estimated efficacy of the different questing tick sampling methods in this first experimental stage is summarized in Table 3 and Supplementary Table S1. The general model showed statistically significant effects on the probability of tick capture of the method, the cumulative time indicator of space use by hosts, the temperature of the soil in the 24 h prior to sampling, and the density of ticks in the plots (Table 4). ASCs were the most effective of the methods for capturing marked adult *H. lusitanicum* ticks (Table 4; Supplementary Tables S2–S11) at any tick density and in all 3 habitats (Supplementary Table S2).

**Table 3.** Average capture efficacy (in percentage), efficacy range, and associated standard error (SE) for each of the studied tick capture methods and efforts throughout habitat type in experimental stage 1. Efforts were low (1 m drags/flags, 4 ASC, and 1 CO_2_ trap per plot), medium (5 m drags/flags, 8 ASC, and 2 CO_2_ traps per plot), and high (10 m drags/flags and 12 ASC)

Habitat		BD^a^			BF^b^			ASC^c^			CDT^d^	
		1 m	5 m	10 m	1 m	5 m	10 m	4	8	12	1	2
Grassland	Average	2.6	1.1	1.7	2.6	1.5	4.2	12.8	15.1	18.7	6.0	8.5
	*Range*	*0–19*	*0–11*	*0–8*	*0–19*	*0–17*	*0–19*	*0–72*	*0–44*	*0–67*	*0–14*	*0–37*
	*SE*	*5.5*	*3.1*	*2.8*	*5.5*	*4.6*	*5.7*	*17.5*	*14.3*	*17.2*	*4.7*	*9.9*
Shrubland	Average	0.0	0.0	0.5	0.0	0.0	0.3	19.1	19.2	22.3	7.1	10.5
	*Range*	*0–0*	*0–0*	*0–4*	*0–0*	*0–0*	*0–3*	*0–64*	*0–44*	*11–50*	*0–37*	*0–50*
	*SE*	*0.0*	*0.0*	*1.2*	*0.0*	*0.0*	*0.9*	*22.0*	*13.1*	*9.8*	*10.3*	*13.9*
Forest	Average	0.0	0.0	0.7	0.0	0.0	1.0	17.0	18.9	23.4	9.7	18.3
	*Range*	*0–0*	*0–0*	*0–4*	*0–0*	*0–0*	*0–7*	*0–56*	*0–78*	*0–56*	*0–22*	*0–33*
	*SE*	*0.0*	*0.0*	*1.4*	*0.0*	*0.0*	*2.1*	*15.0*	*21.1*	*16.4*	*7.8*	*10.4*
All	Average	**0.9**	**0.4**	**1.0**	**0.9**	**0.5**	**1.8**	**16.3**	**17.7**	**21.4**	**7.6**	**12.4**
	Range	**0** *–* **19**	**0** *–* **11**	**0** *–* **8**	**0** *–* **19**	**0** *–* **17**	**0** *–* **19**	**0** *–* **72**	**0** *–* **78**	**0** *–* **67**	**0** *–* **37**	**0** *–* **50**
	SE	**3.3**	**1.8**	**2.0**	**3.3**	**2.7**	**3.9**	**18.2**	**16.3**	**14.6**	**7.9**	**12.1**

^a^Blanket dragging.

^b^Blanket flagging.

^c^Absolute surface count.

^d^CO_2_ traps.

**Table 4.** Output of general and method-specific models including model coefficient estimates and associated standard error (SE), the statistic (*z*), and the *P*-value (*P*).

Model	Predictor	Estimate	SE	z	*P*
General	*Intercept*	*−6.4321*	*0.6899*	*−9.323*	***
	Method				***
	BD	Ref.^a^			
	BF	0.3307	0.2489	1.329	>0.05
	ASC	3.3489	0.1946	17.205	***
	CDT	2.5773	0.2029	12.703	***
	Host^b^	−0.1054	0.0509	−2.072	*
	St^c^	1.3080	0.5145	2.542	*
	Td^d^				***
	d1	Ref.^a^			
	d2	0.3474	0.1635	2.125	*
	d3	0.1105	0.1583	0.698	>0.05
	d4	0.1712	0.1530	1.119	>0.05
	d5	−0.2893	0.1546	−1.871	>0.05
Blanket dragging (BD)	*Intercept*	*−20.8465*	*9.7144*	*−2.146*	*
	Habitat				***
	Grassland	Ref.^a^			
	Shrubland	−2.3197	0.6795	−3.414	***
	Forest	−0.9691	0.6655	−1.456	>0.05
	Effort				>0.05
	Low	Ref.^a^			
	Medium	−1.1710	0.8480	−1.381	>0.05
	High	−0.7000	0.7710	−0.908	>0.05
	Host^b^	0.5963	0.3850	1.549	>0.05
	St^c^	12.9033	7.4205	1.739	>0.05
	Td^d^				>0.05
	d1	Ref.^a^			
	d2	−0.2933	0.9220	−0.318	>0.05
	d3	−1.8104	1.2308	−1.471	>0.05
	d4	0.4995	0.7687	0.650	>0.05
	d5	−0.1076	0.7903	−0.136	>0.05
Blanket flagging (BF)	*Intercept*	−*37.9303*	*17.6313*	−*0.053*	*
	Habitat				***
	Grassland	Ref.^a^			
	Shrubland	−4.1252	1.0478	−3.937	***
	Forest	0.1280	0.8722	0.147	>0.05
	Effort				>0.05
	Low	Ref.^a^			
	Medium	−1.0877	0.6433	−1.691	>0.05
	High	−2.0493	1.2798	−1.601	>0.05
	Host^b^	1.8355	0.5818	3.155	**
	St^c^	25.1735	13.4825	1.867	>0.05
	Td^d^				*
	d1	Ref.^a^			
	d2	0.5831	0.8163	0.714	>0.05
	d3	−0.7087	0.9242	−0.767	>0.05
	d4	0.9080	0.7565	1.200	>0.05
	d5	−0.2294	0.8023	−0.286	>0.05
Absolute surface count (ASC)	*Intercept*	−*4.2331*	*1.7453*	−*2.425*	*
	Habitat				***
	Grassland	Ref.^a^			
	Shrubland	0.8203	0.1220	6.724	***
	Forest	0.2170	0.1425	1.523	>0.05
	Effort				***
	Low	Ref.^a^			
	Medium	0.6650	0.1449	4.590	***
	High	0.7131	0.1775	4.017	***
	Host^b^	−0.6268	0.0935	−6.701	***
	St^c^	1.9173	1.3411	1.430	>0.05
	Td^d^				***
	d1	Ref.^a^			
	d2	0.5201	0.2071	2.511	*
	d3	0.1958	0.2012	0.973	>0.05
	d4	0.1773	0.1956	0.906	>0.05
	d5	−0.0835	0.1946	−0.429	>0.05
CO_2_-trapping (CDT)	*Intercept*	*5.3560*	*2.1220*	*2.524*	*
	Habitat				*
	Grassland	Ref.^a^			
	Shrubland	0.5512	0.2080	2.649	**
	Forest	0.2625	0.2229	1.177	>0.05
	Effort				*
	Low	Ref.^a^			
	Medium	0.4942	0.1932	2.558	*
	Host^b^	−0.4944	0.1904	−2.597	**
	St^c^	−0.6104	0.1620	−3.768	***
	Td^d^				***
	d1	Ref.^a^			
	d2	−0.0000	0.3098	0.000	>0.05
	d3	0.1223	0.2889	0.423	>0.05
	d4	−0.0323	0.2835	−0.114	>0.05
	d5	−0.9477	0.3032	−3.126	**

^a^Reference class in categorical predictors.

^b^Host space usage.

^c^Soil temperature.

^d^Tick density in the plots.

*P < 0.05; **P < 0.01; ***P < 0.001

Blanket dragging and BF were similar in efficacy (Table 4; Supplementary Table S3) and produced the lowest capture efficiency of the 4 methods analyzed (Table 3; Supplementary Table S2). Both BD and BF were more effective in grassland habitats (Table 4; Supplementary Tables S5 and S7), regardless of tick density (Supplementary Tables S5 and S7), where the highest capture efficiencies were 2.6% and 4.2%, respectively (Table 3). In shrubland, the efficiency achieved with these methods was very low (Table 3; Supplementary Tables S4 and S6). Increasing sampling effort had no effect on tick capture probability of both BD and BF methods (Supplementary Tables S5 and S7). Blanket dragging efficiency was neither affected by host space use nor by soil temperature, while blanket flagging efficiency was boosted with host usage and not influenced by soil temperature (Table 4).

The effectiveness of the ASC method ranged from 12.8 % to 23.4 % (Table 3; Supplementary Table S9). However, tick density had a statistically significant effect on the capture efficiency of the ASC method, with the highest capture probability observed in plots with 2 ticks/m^2^ (Table 4; Supplementary Table S9). In terms of sampling effort, the best results for ASC were obtained with increasing effort in all habitats (Table 3; Supplementary Tables S8 and S9), and the variations were statistically significant (Table 4). However, the differences between medium and high effort were not statistically significant for the ASC method (Supplementary Table S9). The habitat statistically influenced the probability of tick capture of the ASC method (Table 4), with the highest efficiencies occurring in shrubland (Supplementary Table S9). In grassland, efficiencies of 12.8% were obtained for low effort, 15.1% for medium effort, and 18.7% for high effort. In shrubland, the efficiency was 19.1% for low effort, 19.2% for medium effort, and 22.3% for high effort, respectively. Finally, in the forest, efficiencies of 17.0%, 18.9%, and 23.4% were obtained for low, medium, and high efforts, respectively. A statistically significant negative effect of tick hosts on ASC capture efficiency was observed (Table 4), while no effect of soil temperature was found.

Capturing efficiencies for CDT were slightly lower than those of ASC (Supplementary Tables S2 and S3) and ranged from 6.0% to 18.3% (Table 3; Supplementary Table S1), with slight variations with increasing tick density (Table 4)—lower efficiency in the plots with five ticks/m^2^ (Supplementary Table S11)—and with higher efficiencies in shrubland with respect grassland (Supplementary Table S11). Deploying 2 traps instead of one per plot improved the capture efficiency of the CDT method. Cumulative host space use and soil temperature had statistically significant negative effects on the efficacy of the CDT method (Table 4).

The camera traps captured 380 visits of wild ungulates to the study plots throughout this first stage of the experiment. Of the visits, 85.3% were red deer (*n* = 324), 10.5% were wild boar (*n* = 40), and 4.2% were roe deer (*n* = 16). Detailed information on the number of visits and images taken by camera traps by wild ungulate species and habitat is detailed in Supplementary Table S12.

There were no tick deaths in the controlled natural mortality estimation containers during the entire experiment. All ticks were in perfect condition at the end of this first experimental stage, so the fluorescent dye was harmless, as expected.

### Second Stage of the Experiment

The Pearson correlation matrix between the continuous predictors estimated at this stage of the experiment is provided in Supplementary Fig. S5. The results of the cumulative efficacy in this second experimental stage are summarized in Table 5 and Supplementary Table S13. The overall model showed statistically significant differences in the capture efficiency of the 4 methods tested and the influence of specific tick densities in the plots on the probability of capture (Table 6; Supplementary Tables S14 and S15). No effect of accumulated host space use or soil temperature in the 24 h prior to sampling was evident (Table 6). As in the first experiment, the method with the highest cumulative capture efficacy was ASC, followed by CDT, demonstrating once again that the least effective methods for capturing *H. lusitanicum* adults were blanket dragging and flagging (Table 6; Supplementary Tables S14–S23).

**Table 5.** Cumulative capture average efficacy (in percentage), efficacy range and associated standard error (SE) on day 3 of consecutive 3-day surveys performed in stage 2 of the experiment. Results are shown per capture method and effort throughout habitat type. Efforts were low (1 m drags/flags, 4 ASC, and 1 CO_2_ trap per plot), medium (5 m drags/flags and 8 ASC), and high (10 m drags/flags and twelve ASC)

Habitat		BD^a^			BF^b^			ASC^c^			CDT^d^
		1 m	5 m	10 m	1 m	5 m	10 m	4	8	12	1
	Average	1.0	5.3	0.0	1.0	0.0	0.3	51.7	51.0	32.7	29.0
Grassland	*Range*	*0–3*	*4–6*	*0–0*	*0–3*	*0–0*	*0–6*	*41–58*	*31–78*	*7–69*	*26–33*
	*SE*	*1.7*	*1.2*	*0.0*	*1.7*	*0.0*	*3.1*	*9.3*	*24.3*	*32.3*	*3.6*
Shrubland	Average	1.3	1.0	2.0	1.3	3.3	7.0	72.3	69.7	51.3	9.3
	*Range*	*0–4*	*0–3*	*0–6*	*0–4*	*0–6*	*6–8*	*56–83*	*56–83*	*26–72*	*3–19*
	*SE*	*2.3*	*1.7*	*3.5*	*2.3*	*3.1*	*1.0*	*14.4*	*13.5*	*23.4*	*8.5*
Forest	Average	1.0	2.0	2.3	1.0	0.0	0.0	74.7	45.0	36.3	27.3
	*Range*	*0–3*	*0–6*	*0–4*	*0–3*	*0–0*	*0–0*	*67–83*	*39–52*	*26–44*	*17–37*
	*SE*	*1.7*	*3.5*	*2.1*	*1.7*	*0.0*	*0.0*	*8.0*	*6.7*	*9.3*	*10.0*
**All**	**Average**	**1.1**	**2.8**	**1.4**	**1.1**	**1.1**	**3.4**	**66.2**	**55.2**	**40.1**	**21.9**
	***Range***	***0*** *–* ***4***	***0*** *–* ***6***	***0*** *–* ***6***	***0*** *–* ***4***	***0*** *–* ***6***	***0*** *–* ***8***	***41*** *–* ***83***	***31*** *–* ***83***	***7*** *–* ***72***	***3*** *–* ***37***
	***SE***	***1.7***	***2.8***	***2.3***	***1.7***	***2.3***	***3.4***	***14.5***	***18.1***	***22.2***	***11.7***

^a^Blanket dragging.

^b^Blanket flagging.

^c^Absolute surface count.

^d^CO_2_ traps.

**Table 6.** Output of general and method-specific models for the cumulative efficacy between the different capture methods including model coefficient estimates and associated standard error (SE), the statistic (*z*), and the *P*-value (*P*)

Model	Predictor	Estimate	SE	z	*p*
General	*Intercept*	*−0.7904*	*1.6303*	*−0.485*	*>0.05*
	Method				***
	BD	Ref.^a^			
	BF	0.1892	0.3837	0.493	>0.05
	ASC	4.1810	0.2905	14.394	***
	CDT	2.6365	0.3262	8.082	***
	Host^b^	*−*0.1368	0.0805	*−*1.699	>0.05
	St^c^	*−*2.3017	1.2085	*−*1.905	>0.05
	Td^d^				*
	d2	Ref.^a^			
	d3	*−*0.2905	0.1757	*−*1.654	>0.05
	d4	0.1527	0.1645	0.928	>0.05
Blanket dragging (BD)	*Intercept*	*−60.3400*	*40.6980*	*−1.284*	*>0.05*
	Habitat				>0.05
	Grassland	Ref.^a^			
	Shrubland	3.4110	3.6050	0.946	>0.05
	Forest	4.2280	4.2410	0.997	>0.05
	Effort				>0.05
	Low	Ref.^a^			
	Medium	2.604	2.1330	1.221	>0.05
	High	*−*0.9400	1.6860	*−*0.558	>0.05
	Host^b^	0.3568	0.8145	0.438	>0.05
	St^c^	39.320	32.580	1.207	>0.05
	Td^d^				>0.05
	d2	Ref.^a^			
	d3	*−*0.0000	0.9208	0.000	>0.05
	d4	0.7108	0.7989	0.890	>0.05
Blanket flagging (BF)	*Intercept*	*11.7435*	*25.6066*	*0.459*	*>0.05*
	Habitat				**
	Grassland	Ref.^a^			
	Shrubland	1.3721	2.0330	0.675	>0.05
	Forest	*−*1.5410	1.9014	*−*0.810	>0.05
	Effort				>0.05
	Low	Ref.^a^			
	Medium	*−*1.2935	1.5709	*−*0.823	>0.05
	High	1.2277	0.7817	1.571	>0.05
	Host^b^	0.7496	1.1854	0.632	>0.05
	St^c^	*−*14.0903	18.3152	*−*0.769	>0.05
	Td^d^				>0.05
	d2	Ref.^a^			
	d3	1.2337	1.1051	1.116	>0.05
	d4	1.5489	1.0632	1.457	>0.05
Absolute surface count (ASC)	*Intercept*	*5.7305*	*4.5948*	*1.247*	*>0.05*
	Habitat				***
	Grassland	Ref.^a^			
	Shrubland	0.6297	0.2999	1.247	*
	Forest	*−*0.1409	0.3732	*−*0.378	>0.05
	Effort				***
	Low	Ref.^a^			
	Medium	*−*0.5880	0.2671	*−*2.201	*
	High	*−*0.9352	0.2017	*−*4.637	***
	Host^b^	*−*0.0751	0.1101	*−*0.682	>0.05
	St^c^	*−*3.7624	3.3087	*−*1.137	>0.05
	Td^d^				***
	d2	Ref.^a^			
	d3	*−*0.5818	0.2131	*−*2.730	**
	d4	0.1237	0.2029	0.610	>0.05
CO_2_*-*trapping (CDT)	*Intercept*	*−0.9270*	*0.3917*	*−2.367*	*
	Habitat				**
	Grassland	Ref.^a^			
	Shrubland	*−*1.5081	0.4666	*−*3.232	**
	Forest	*−*0.1874	0.3539	*−*0.530	>0.05
	Td^d^				>0.05
	d2	Ref.^a^			
	d3	0.3989	0.4320	0.923	>0.05
	d4	*−*0.1894	0.4320	*−*0.438	>0.05

^a^Reference class in categorical predictors.

^b^Host space usage.

^c^Soil temperature.

^d^Tick density in the plots.

*P < 0.05; **P < .01; ***P < .001.

Repetition on 3 consecutive days for each habitat replicate in the ASC and CDT methods resulted in higher capture efficiency regardless of sampling effort and tick density (Table 5) with respect to point sampling (Table 3), while it only slightly improved the capture efficiency of BD and BF in shrubland and forest when compared to point sampling. However, increasing sampling effort from low to high did not provide higher cumulative capture efficiency in any of the methods tested, while it negatively affected the capture probability of the ASC method (Table 6; Supplementary Table S21).

The cumulative efficiency of BD on the third day of the 3 consecutive sampling days was slightly higher, but not statistically significant, while no effect of effort on capture efficiency was found. Flagging performed poorly in forested environments, but higher flagging effort showed a positive, although not statistically significant, effect on cumulative capture efficiency. The highest efficiency for the BF method (7%) was obtained for the high effort in shrubland (Table 5), but it did not statistically differ from the efficacies of the method at lower sampling efforts (Supplementary Table S19).

The accumulated efficacy of the ASC method was influenced by the habitat, sampling effort, and tick density (Table 6) but not by host space usage and soil temperature. ASC performed better in shrubland habitats, where we observed statistically significant positive differences with respect to other environments (Table 6; Supplementary Tables S20 and S21). In contrast, increasing survey effort had a statistically significant negative influence on efficacy on the third day (Table 6; Supplementary Tables S20 and S21). The highest capture probability of marked *H. lusitanicum* ticks with the ASC method was observed in the plots with the lowest tick density, 2 ticks/m^2^ (Table 6; Supplementary Table S21).

The CDT method performed better in open grasslands than in shrublands and woodlands, with a statistically significant decrease in efficacy in shrublands (Table 6; Supplementary Table S22). Variations in tick density did not affect CDT efficiency (Table 6; Supplementary Table S22).

The camera traps captured 144 wild ungulate visits throughout the second stage of the experiment, 141 (97.9%) red deer, 2 (1.4%) wild boar, and 1 (0.7%) roe deer. Detailed information is given in Supplementary Table S1.

As in experimental stage 1, natural tick mortality in the containers deployed in the study plots was zero both for dye-marked and unmarked *H. lusitanicum* adult ticks.

## Discussion

Several hematophagous arthropods are vectors of infectious pathogens that cause serious animal and human diseases (Ruiz-Fons et al. 2012, 2014, Cuadrado-Matías et al. 2022), so accurately estimating vector population size is essential for epidemiological inference of vector-borne diseases. Without an accurate estimate of the actual population size, epidemiologists cannot estimate disease/pathogen transmission risks robustly enough to support sound prevention recommendations. Most wildlife epidemiological studies employ population size approximations (Acevedo et al. 2008). Estimates of the population size of questing ticks are conditioned by the efficacy of the selected method (which depends on the behavioral ecology of the species/group). This limitation of the efficiency could be due to the method’s inability to count the total population and to capture a representative sample from which to infer population size (Bugmyrin and Gorbach 2022), or because the capture efficiency of the method is modulated by tick habitats, e.g., variations in vegetation height and density (Ruiz-Fons and Gilbert 2010). No study had previously attempted to analyze the efficacy of exophilic tick capture methods for estimating the true density of species of the genus *Hyalomma*. This experimental trial estimates capture efficiency values for both existing and novel tick capture methods, as well as for particular variations in the habitat in which *H. lusitanicum* ticks live.

We selected the model study, *H. lusitanicum*, because it is considered the most relevant vector of CCHFV in the western Mediterranean (Sánchez-Seco et al. 2021, Cuadrado-Matías et al. 2022). Our results suggest that ASC is the method of choice for population monitoring of questing *H. lusitanicum* adults. In addition, we also found that serial consecutive repetitions of ASC could significantly improve the efficiency of population estimation. However, an increase in sampling effort had negative effects on capture probability in the second stage of our experiment, with lower efficacy for 12 counts than for 8 and 4 counts when counts are repeated and cumulative efficacy is estimated. We do not know the cause of this lower efficacy, especially in the absence of a clear effect of cumulative use of space by hosts that could have reduced the number of ticks deposited on the plots over time and with the exhaustive process of monitoring tick movements from their plots implemented. Although overall, soil temperature had no effect on the probability of capture, relative humidity was the lowest on the sampling days of the second experiment (Supplementary Fig. S3), perhaps circumstantially affecting tick activity. Future experiments may better clarify the relationship between sampling effort and cumulative efficacy for this method. We sampled consecutively on a daily basis, but most likely repeating sampling over a single day would yield similar cumulative capture efficiency results while reducing the time required. Serial sampling would most likely benefit from the activation of nonquesting ticks by previous sampling passages. However, along with our recommendation, we must consider that the efficacy of the ASC method, as estimated from our experimental approach, may not be completely realistic as ticks were highly spatially aggregated in our experimental plots. *Hyalomma lusitanicum* ticks naturally aggregate at higher densities than tested in our study in particular sites at larger spatial scales, i.e., at those at which tick densities are estimated for ecological and epidemiological studies (Salomon et al. 2020). This occurs because nymphs detach from hosts at locations where hosts aggregate, e.g., ungulates seeking cooling shade under solitary evergreen oaks in patched savannah-type habitats, and that was indeed the reasoning for designing the ASC method. However, aggregation may only occur in limited patches within their local spatial distribution range, suggesting that the large-scale efficacy of the ASC method should be corroborated before recommending it as the population monitoring tool of choice for questing *Hyalomma* spp. ticks. Furthermore, the ASC method requires a great effort if it is to be employed on large patches of land that also needs to be considered and dimensioned before recommending it.

Blanket dragging and flagging were found to be of similarly low efficacy, which a priori would make it a low recommendable method to capture adult *H. lusitanicum*. This agrees with previous findings in different tick species (Tälleklint-Eisen and Lane 2000, Nyrhilä et al. 2020). At first glance, this would seem realistic if one expects predominantly hunting rather than ambushing behavior for the tick species studied (Valcárcel et al. 2020). However, our field experience employing BD for serial surveys of questing *H. lusitanicum* ticks shows that the method is useful for capturing them at large spatial scales, although no estimate of efficacy has been made to estimate the proportion of the actual tick population it captures. In fact, we conducted blanket flagging over large areas of the study area and collected several thousand *H. lusitanicum* as they quested for hosts on vegetation spikes, but we did not estimate what proportion of questing ticks were collected. This again suggests that the methods studied should also be tested at larger spatial scales and in different habitat structures to test their efficacy, but also their logistic cost-effectiveness. In any case, our estimates provide a basis for adjusting BD and BF estimates of adult *H. lusitanicum* to the actual population size that could be useful for epidemiological modeling of transmitted pathogens.

Carbon dioxide-based traps, although an efficient tool for capturing *H. lusitanicum* adults as demonstrated for other hunter ticks (Kensinger and Allan 2011), are expensive to use, as they need to be baited with dry ice. Previous experimental tests conducted in QM with different CO_2_ sources showed that only dry ice was efficient to capture *H. lusitanicum* and *Rhipicephalus bursa* ticks (the authors, unpublished). Increasing the effort for larger study areas would therefore require a large number of CO_2_ traps and, consequently, a higher economic cost. We did not estimate the effective distance of attraction of the CO_2_ traps to estimate the denominator soil surface from which ticks can be attracted and calculate a density value, but we are confident that it covered the entire surface of the 3 × 3 m plots. In no case did they attract ticks marked with another color from neighboring 3 × 3 m plots in any of the replicates in the 2 stages of the experiment, suggesting that their spatial range of capture is at least below 9 m, which is the minimum distance between the center of 2 neighboring plots where the marked ticks were deployed.

The main conclusion we can draw from our results is that there is great variability in the efficacy of the methods traditionally used to capture free-living ticks when applied to capture adult *H. lusitanicum*. As would be expected for a species that exhibits a hunter–host-seeking strategy, actively searching for adult *H. lusitanicum* ticks (and perhaps other ticks of *Hyalomma* spp.) was more efficient at capturing the ticks than those methods that are more appropriate for ambush ticks. However, our results demonstrate that adult *H. lusitanicum* ticks also climb vegetation to passively find hosts. Perhaps combining methods to capture *H. lusitanicum* both actively and passively would be advisable to improve the estimation of population size. In this study, we have laid the groundwork for estimating the actual population size of *Hyalomma* spp. in Mediterranean ecosystems, which in the future will allow us to obtain greater robustness and accuracy in predictive epidemiological models. Further, accurate population size estimation tools would also allow robust monitoring of tick population changes. Methods that have experimentally demonstrated higher capture efficiency may be complicated or expensive to apply at large spatiotemporal sampling scales. Thus, the main recommendation is to choose the method(s) that best fits the objectives of the study and use the estimated efficacy ranges to transform abundance estimates into values that more closely approximate the true density of active questing ticks. Given the influence of sampling habitat, it is essential to take this factor into account in transforming abundance estimates.

## Supplementary Material

tjad127_suppl_Supplementary_Figures_S1-S5_Tables_S1-S23

## Data Availability

Data supporting the conclusions of this study are included in the manuscript. If further clarification is needed, requests may be directed to the corresponding author.
