# Supplementary material for: Testing the efficiency of capture methods for questing Hyalomma lusitanicum (Acari: Ixodidae), a vector of Crimean-Congo hemorrhagic fever virus
Source: J Med Entomol. 2023 Sep 13;61(1):152–65. doi: 10.1093/jme/tjad127 (PMC10784776; doi:10.1093/jme/tjad127)
Supplement: tjad127_suppl_Supplementary_Figures_S1-S5_Tables_S1-S23 [file tjad127_suppl_supplementary_figures_s1-s5_tables_s1-s23.docx]

**Supplementary Table S1.** Average capture efficacy (%) for each of the studied tick capture methods and efforts throughout habitat type and tick density in experimental stage 1. Efforts were low (1m drags/flags, four ASC, and 1 CO_2_ trap per plot), medium (5m drags/flags, eight ASC, and 2 CO_2_ traps per plot), and high (10m drags/flags and twelve ASC).

|  |  | **BD^a^** | | | **BF^b^** | | | **ASC^c^** | | | **CDT^d^** | |
| --- | --- | --- | --- | --- | --- | --- | --- | --- | --- | --- | --- | --- |
|  |  | 1m | 5m | 10m | 1m | 5m | 10m | 4 | 8 | 12 | 1 | 2 |
|  | Ticks/m^2^ |  |  |  |  |  |  |  |  |  |  |  |
| Grassland | 1 | 3.7 | 3.7 | 0.0 | 3.7 | 0.0 | 3.7 | 3.7 | 14.8 | 29.6 | 7.4 | 7.4 |
|  | 2 | 1.9 | 1.9 | 1.9 | 1.9 | 5.6 | 5.6 | 33.3 | 24.1 | 16.7 | 9.3 | 11.1 |
|  | 3 | 0.0 | 0.0 | 0.0 | 0.0 | 0.0 | 1.2 | 16.1 | 11.1 | 17.3 | 3.7 | 16.1 |
|  | 4 | 6.5 | 0.0 | 4.6 | 6.5 | 1.9 | 7.4 | 5.6 | 8.3 | 16.7 | 7.4 | 3.7 |
|  | 5 | 0.7 | 0.0 | 2.2 | 0.7 | 0.0 | 3.0 | 5.2 | 17.0 | 13.3 | 2.2 | 4.4 |
|  | *Average* | *2.6* | *1.1* | *1.7* | *2.6* | *1.5* | *4.2* | *12.8* | *15.1* | *18.7* | *6.0* | *8.5* |
| Shrubland | 1 | 0.0 | 0.0 | 0.0 | 0.0 | 0.0 | 0.0 | 0.0 | 7.4 | 14.1 | 0.0 | 7.4 |
|  | 2 | 0.0 | 0.0 | 0.0 | 0.0 | 0.0 | 0.0 | 27.8 | 25.9 | 33.3 | 1.9 | 25.9 |
|  | 3 | 0.0 | 0.0 | 0.0 | 0.0 | 0.0 | 0.0 | 18.5 | 28.4 | 17.3 | 17.3 | 8.6 |
|  | 4 | 0.0 | 0.0 | 0.9 | 0.0 | 0.0 | 0.9 | 38.9 | 15.7 | 25.9 | 13.0 | 7.4 |
|  | 5 | 0.0 | 0.0 | 1.5 | 0.0 | 0.0 | 0.7 | 10.4 | 18.5 | 20.0 | 3.7 | 3.0 |
|  | *Average* | *0.0* | *0.0* | *0.5* | *0.0* | *0.0* | *0.3* | *19.1* | *19.2* | *22.3* | *7.2* | *10.5* |
| Forest | 1 | 0.0 | 0.0 | 0.0 | 0.0 | 0.0 | 0.0 | 25.9 | 25.9 | 22.2 | 14.8 | 29.6 |
|  | 2 | 0.0 | 0.0 | 0.0 | 0.0 | 0.0 | 0.0 | 18.5 | 16.7 | 20.4 | 7.4 | 11.1 |
|  | 3 | 0.0 | 0.0 | 1.2 | 0.0 | 0.0 | 2.5 | 21.0 | 18.5 | 21.0 | 7.4 | 21.0 |
|  | 4 | 0.0 | 0.0 | 0.0 | 0.0 | 0.0 | 0.9 | 9.3 | 17.6 | 28.7 | 13.9 | 19.4 |
|  | 5 | 0.0 | 0.0 | 2.2 | 0.0 | 0.0 | 1.5 | 10.4 | 15.6 | 24.4 | 4.4 | 10.4 |
|  | *Average* | *0.0* | *0.0* | *0.7* | *0.0* | *0.0* | *1.0* | *17.0* | *18.9* | *23.4* | *9.6* | *18.3* |
| Total | | 0.9 | 0.4 | 1.0 | 0.9 | 0.5 | 1.8 | 16.3 | 17.7 | 21.4 | 7.6 | 12.4 |

**^a^**Blanket dragging; **^b^**Blanket flagging; **^c^**Absolute surface count; **^d^**CO_2_ traps.

**Supplementary Table S2.** Probability of capture of marked ticks as a function of tick density in the experimental plots and capture method estimated (‘lsmeans’ function of the R package ‘emmeans’) from the best fitted general model for the stage 1 of the experiment as presented in Table 4. The probability values (Prob.; scale: 0-1), the standard error of their estimate (SE) and the lower (LCL) and upper (UCL) limits of the estimated interval with 95% confidence are shown for each level of the categorical predictors of the model.

| **Tick density^a^** | **Method^b^** | **Prob.** | **SE** | **LCL** | **UCL** |
| --- | --- | --- | --- | --- | --- |
| d1 | BD | 0.0073 | 0.0017 | 0.0047 | 0.0115 |
|  | BF | 0.0102 | 0.0021 | 0.0068 | 0.0152 |
|  | ASC | 0.1735 | 0.0202 | 0.1375 | 0.2167 |
|  | CDT | 0.0885 | 0.0122 | 0.0672 | 0.1156 |
|  | *All* | *0.0340* | *0.0049* | *0.0256* | *0.0451* |
| d2 | BD | 0.0103 | 0.0021 | 0.0069 | 0.0154 |
|  | BF | 0.0143 | 0.0025 | 0.0101 | 0.0202 |
|  | ASC | 0.2291 | 0.0161 | 0.1991 | 0.2621 |
|  | CDT | 0.1208 | 0.0113 | 0.1003 | 0.1447 |
|  | *All* | *0.0475* | *0.0046* | *0.0392* | *0.0575* |
| d3 | BD | 0.0082 | 0.0016 | 0.0055 | 0.0121 |
|  | BF | 0.0113 | 0.0020 | 0.0081 | 0.0159 |
|  | ASC | 0.1900 | 0.0125 | 0.1667 | 0.2156 |
|  | CDT | 0.0978 | 0.0087 | 0.0820 | 0.1162 |
|  | *All* | *0.0379* | *0.0035* | *0.0317* | *0.0452* |
| d4 | BD | 0.0087 | 0.0017 | 0.0059 | 0.0127 |
|  | BF | 0.0120 | 0.0020 | 0.0087 | 0.0167 |
|  | ASC | 0.1995 | 0.0112 | 0.1784 | 0.2223 |
|  | CDT | 0.1033 | 0.0083 | 0.0881 | 0.1208 |
|  | *All* | *0.0401* | *0.0033* | *0.0342* | *0.0471* |
| d5 | BD | 0.0055 | 0.0011 | 0.0037 | 0.0081 |
|  | BF | 0.0076 | 0.0013 | 0.0055 | 0.0107 |
|  | ASC | 0.1359 | 0.0086 | 0.1199 | 0.1536 |
|  | CDT | 0.0678 | 0.0059 | 0.0571 | 0.0802 |
|  | *All* | *0.0257* | *0.0022* | *0.0217* | *0.0305* |
| *All* | *BD* | *0.0078* | *0.0015* | *0.0054* | *0.0113* |
|  | *BF* | *0.0109* | *0.0018* | *0.0079* | *0.0149* |
|  | *ASC* | *0.1835* | *0.0072* | *0.1698* | *0.1980* |
|  | *CDT* | *0.0941* | *0.0063* | *0.0824* | *0.1073* |

^a^d1=1/m^2^, d2=2/m^2^, d3=3/m^2^, d4=4/m^2^, d5=5/m^2^; ^b^BD: blanket dragging, BF: blanket flagging, ASC: absolute surface count, CDT: CO_2_ traps.

**Supplementary Table S3.** Odds ratios resulting from the paired comparison of the different levels of the categorical predictors (capture method and tick density in the experimental plots) of the model with best overall fit of stage 1 of the experiment (Table 4) estimated using the 'lsmeans' function of the 'emmeans' package of R. The odds ratio (OR) value of each paired comparison, the standard error (SE) of that value, the statistic (z) and the p-value (*p*) are presented.

| **Predictor** | **OR** | **SE** | **z** | ***p^c^*** |
| --- | --- | --- | --- | --- |
| Method^a^ |  |  |  |  |
| BD *vs.* BF | 0.7184 | 0.1788 | -1.3290 | >0.05 |
| BD *vs.* ASC | 0.0351 | 0.0068 | -17.2050 | *** |
| BD *vs.* CDT | 0.0760 | 0.0154 | -12.7030 | *** |
| BF *vs.* ASC | 0.0489 | 0.0082 | -18.0950 | *** |
| BF *vs.* CDT | 0.1058 | 0.0187 | -12.7330 | *** |
| ASC *vs.* CDT | 2.1634 | 0.1815 | 9.1990 | *** |
| Tick density^b^ |  |  |  |  |
| d1 *vs.* d2 | 0.7070 | 0.1155 | -2.1520 | >0.05 |
| d1 *vs.* d3 | 0.8950 | 0.1417 | -0.6980 | >0.05 |
| d1 *vs.* d4 | 0.8430 | 0.1289 | -1.1190 | >0.05 |
| d1 *vs.* d5 | 1.3360 | 0.2065 | 1.8710 | >0.05 |
| d2 *vs.* d3 | 1.2670 | 0.1471 | 2.0410 | >0.05 |
| d2 *vs.* d4 | 1.1930 | 0.1297 | 1.6210 | >0.05 |
| d2 *vs.* d5 | 1.8900 | 0.2099 | 5.7330 | *** |
| d3 *vs.* d4 | 0.9410 | 0.0948 | -0.6030 | ** |
| d3 *vs.* d5 | 1.4920 | 0.1540 | 3.8710 | >0.05 |
| d4 *vs.* d5 | 1.5850 | 0.1504 | 4.8520 | *** |

^a^BD: blanket dragging, BF: blanket flagging, ASC: absolute surface count, CDT: CO_2_ traps;^b^d1=1/m^2^, d2=2/m^2^, d3=3/m^2^, d4=4/m^2^, d5=5/m^2^; ^c^*p<.05, **p<.01, ***p<.001.

**Supplementary Table S4.** Probability of capture of marked ticks as a function of habitat and effort estimated (‘lsmeans’ function of the R package ‘emmeans’) from the best fitted model for the blanket dragging method for the stage 1 of the experiment as presented in Table 4. The probability values (Prob.; scale: 0-1), the standard error of their estimate (SE) and the lower (LCL) and upper (UCL) limits of the estimated interval with 95% confidence are shown for each level of the categorical predictors of the model.

| **Habitat** | **Effort^a^** | **Prob.** | **SE** | **LCL** | **UCL** |
| --- | --- | --- | --- | --- | --- |
| Grassland | low | 0.0132 | 0.0055 | 0.0058 | 0.0295 |
|  | medium | 0.0041 | 0.0032 | 0.0009 | 0.0186 |
|  | high | 0.0066 | 0.0053 | 0.0014 | 0.0311 |
|  | *All* | *0.0071* | *0.0030* | *0.0031* | *0.0162* |
| Shrubland | low | 0.0013 | 0.0010 | 0.0003 | 0.0056 |
|  | medium | 0.0004 | 0.0004 | 0.0001 | 0.0027 |
|  | high | 0.0007 | 0.0008 | 0.0001 | 0.0063 |
|  | *All* | *0.0007* | *0.0016* | *0.0008* | *0.0089* |
| Forest | low | 0.0050 | 0.0035 | 0.0013 | 0.0196 |
|  | medium | 0.0016 | 0.0016 | 0.0002 | 0.0115 |
|  | high | 0.0025 | 0.0017 | 0.0007 | 0.0094 |
|  | *All* | *0.0027* | *0.0016* | *0.0008* | *0.0089* |
| *All* | *low* | *0.0044* | *0.0019* | *0.0019* | *0.0104* |
|  | *medium* | *0.0014* | *0.0011* | *0.0003* | *0.0067* |
|  | *high* | *0.0022* | *0.0017* | *0.0005* | *0.1009* |

^a^low: 1 m drag/plot, medium: 5 m drag/plot, high: 10 m drag/plot.

**Supplementary Table S5.** Odds ratios resulting from the paired comparison of the different levels of the categorical predictors (effort, habitat, and tick density in the experimental plots) of the blanket dragging-specific model with best fit of stage 1 of the experiment (Table 4) estimated using the 'lsmeans' function of the 'emmeans' package of R. The odds ratio (OR) value of each paired comparison, the standard error (SE) of that value, the statistic (z) and the p-value (*p*) are presented.

| **Predictor** | **OR** | **SE** | **z** | ***p^c^*** |
| --- | --- | --- | --- | --- |
| Effort^a^ |  |  |  |  |
| low *vs.* medium | 3.2250 | 2.7400 | 1.3810 | >0.05 |
| low *vs.* high | 2.0140 | 1.5500 | 0.9080 | >0.05 |
| medium *vs.* high | 0.6240 | 0.7200 | -0.4080 | >0.05 |
| Habitat |  |  |  |  |
| grassland *vs.* shrubland | 10.1730 | 6.9120 | 3.4140 | ** |
| grassland *vs.* forest | 2.6350 | 1.7540 | 1.4560 | >0.05 |
| shrubland *vs.* forest | 0.2590 | 0.2590 | -1.3480 | >0.05 |
| Tick density^b^ |  |  |  |  |
| d1 *vs.* d2 | 1.3408 | 1.2360 | 0.3180 | >0.05 |
| d1 *vs.* d3 | 6.1131 | 7.5240 | 1.4710 | >0.05 |
| d1 *vs.* d4 | 0.6068 | 0.4660 | -0.6500 | >0.05 |
| d1 *vs.* d5 | 1.1136 | 0.8800 | 0.1360 | >0.05 |
| d2 *vs.* d3 | 4.5592 | 5.3280 | 1.3090 | >0.05 |
| d2 *vs.* d4 | 0.4526 | 0.2930 | -1.2250 | >0.05 |
| d2 *vs.* d5 | 0.8305 | 0.5590 | -0.2760 | >0.05 |
| d3 *vs.* d4 | 0.0993 | 0.1030 | -2.2190 | >0.05 |
| d3 *vs.* d5 | 0.1822 | 0.1930 | -1.6110 | >0.05 |
| d4 *vs.* d5 | 1.8352 | 0.8060 | 1.3820 | >0.05 |

^a^low: 1 m drag/plot, medium: 5 m drag/plot, high: 10 m drag/plot;^b^d1=1/m^2^, d2=2/m^2^, d3=3/m^2^, d4=4/m^2^, d5=5/m^2^;^c^*p<.05, **p<.01, ***p<.001.

**Supplementary Table S6.** Probability of capture of marked ticks as a function of habitat and effort estimated (‘lsmeans’ function of the R package ‘emmeans’) from the best fitted model for the blanket flagging method for the stage 1 of the experiment as presented in Table 4. The probability values (Prob.; scale: 0-1), the standard error of their estimate (SE) and the lower (LCL) and upper (UCL) limits of the estimated interval with 95% confidence are shown for each level of the categorical predictors of the model.

| **Habitat** | **Effort^a^** | **Prob.** | **SE** | **LCL** | **UCL** |
| --- | --- | --- | --- | --- | --- |
| Grassland | low | 0.0171 | 0.0065 | 0.0081 | 0.0359 |
|  | medium | 0.0058 | 0.0033 | 0.0019 | 0.0173 |
|  | high | 0.0022 | 0.0031 | 0.0002 | 0.0318 |
|  | *All* | *0.0061* | *0.0036* | *0.0019* | *0.0193* |
| Shrubland | low | 0.0003 | 0.0003 | 0.0000 | 0.0027 |
|  | medium | 0.0001 | 0.0001 | 0.0000 | 0.0012 |
|  | high | 0.0000 | 0.0000 | 0.0000 | 0.0028 |
|  | *All* | *0.0000* | *0.0002* | *0.0000* | *0.0018* |
| Forest | low | 0.0194 | 0.0156 | 0.0039 | 0.0902 |
|  | medium | 0.0066 | 0.0058 | 0.0012 | 0.0361 |
|  | high | 0.0025 | 0.0021 | 0.0005 | 0.0123 |
|  | *All* | *0.0069* | *0.0036* | *0.0025* | *0.0192* |
| *All* | *low* | *0.0046* | *0.0020* | *0.0019* | *0.0109* |
|  | *medium* | *0.0015* | *0.0010* | *0.0005* | *0.0052* |
|  | *high* | *0.0006* | *0.0008* | *0.0000* | *0.0091* |

^a^low: 1 m flag/plot, medium: 5 m flag/plot, high: 10 m flag/plot.

**Supplementary Table S7.** Odds ratios resulting from the paired comparison of the different levels of the categorical predictors (effort, habitat, and tick density in the experimental plots) of the blanket flagging-specific model with best fit of stage 1 of the experiment (Table 4) estimated using the 'lsmeans' function of the 'emmeans' package of R. The odds ratio (OR) value of each paired comparison, the standard error (SE) of that value, the statistic (z) and the p-value (*p*) are presented.

| **Predictor** | **OR** | **SE** | **z** | ***p^c^*** |
| --- | --- | --- | --- | --- |
| Effort^a^ |  |  |  |  |
| low *vs.* medium | 2.9700 | 1.9100 | 1.6910 | >0.05 |
| low *vs.* high | 7.7600 | 9.9300 | 1.6010 | >0.05 |
| medium *vs.* high | 2.6200 | 3.4800 | 0.7220 | >0.05 |
| Habitat |  |  |  |  |
| grassland *vs.* shrubland | 61.8795 | 64.8362 | 3.9370 | *** |
| grassland *vs.* forest | 0.8798 | 0.7674 | -0.1470 | >0.05 |
| shrubland *vs.* forest | 0.0142 | 0.0241 | -2.5130 | * |
| Tick density^b^ |  |  |  |  |
| d1 *vs.* d2 | 0.5580 | 0.4560 | -0.7140 | >0.05 |
| d1 *vs.* d3 | 2.0310 | 1.8770 | 0.7670 | >0.05 |
| d1 *vs.* d4 | 0.4030 | 0.3050 | -1.2000 | >0.05 |
| d1 *vs.* d5 | 1.2580 | 1.0090 | 0.2860 | >0.05 |
| d2 *vs.* d3 | 3.6390 | 2.5460 | 1.8470 | >0.05 |
| d2 *vs.* d4 | 0.7230 | 0.3290 | -0.7140 | >0.05 |
| d2 *vs.* d5 | 2.2530 | 1.1900 | 1.5390 | >0.05 |
| d3 *vs.* d4 | 0.1990 | 0.1250 | -2.5720 | >0.05 |
| d3 *vs.* d5 | 0.6190 | 0.4230 | -0.7020 | >0.05 |
| d4 *vs.* d5 | 3.1190 | 1.3140 | 2.6460 | >0.05 |

^a^low: 1 m flag/plot, medium: 5 m flag/plot, high: 10 m flag/plot;^b^d1=1/m^2^, d2=2/m^2^, d3=3/m^2^, d4=4/m^2^, d5=5/m^2^; ^c^*p<.05, **p<.01, ***p<.001.

**Supplementary Table S8.** Probability of capture of marked ticks as a function of habitat and effort estimated (‘lsmeans’ function of the R package ‘emmeans’) from the best fitted model for the absolute surface count method for the stage 1 of the experiment as presented in Table 4. The probability values (Prob.; scale: 0-1), the standard error of their estimate (SE) and the lower (LCL) and upper (UCL) limits of the estimated interval with 95% confidence are shown for each level of the categorical predictors of the model.

| **Habitat** | **Effort^a^** | **Prob.** | **SE** | **LCL** | **UCL** |
| --- | --- | --- | --- | --- | --- |
| Grassland | low | 0.0859 | 0.0101 | 0.0680 | 0.1080 |
|  | medium | 0.1544 | 0.0162 | 0.1252 | 0.1890 |
|  | high | 0.1608 | 0.0235 | 0.1199 | 0.2120 |
|  | *All* | *0.1290* | *0.0109* | *0.1100* | *0.1520* |
| Shrubland | low | 0.1758 | 0.0148 | 0.1487 | 0.2070 |
|  | medium | 0.2932 | 0.0258 | 0.2452 | 0.3460 |
|  | high | 0.3033 | 0.0372 | 0.2357 | 0.3810 |
|  | *All* | *0.2520* | *0.0164* | *0.2220* | *0.2860* |
| Forest | low | 0.1045 | 0.0140 | 0.0801 | 0.1350 |
|  | medium | 0.1849 | 0.0269 | 0.1379 | 0.2440 |
|  | high | 0.1923 | 0.0174 | 0.1605 | 0.2290 |
|  | *All* | *0.1560* | *0.0137* | *0.1310* | *0.1850* |
| *All* | *low* | *0.1170* | *0.0104* | *0.0983* | *0.1390* |
|  | *medium* | *0.2050* | *0.0197* | *0.1692* | *0.2460* |
|  | *high* | *0.2130* | *0.0226* | *0.1721* | *0.2610* |

^a^low: 4 counts/plot, medium: 8 counts/plot, high: 12 counts/plot.

**Supplementary Table S9.** Odds ratios resulting from the paired comparison of the different levels of the categorical predictors (effort, habitat, and tick density in the experimental plots) of the absolute surface count-specific model with best fit of stage 1 of the experiment (Table 4) estimated using the 'lsmeans' function of the 'emmeans' package of R. The odds ratio (OR) value of each paired comparison, the standard error (SE) of that value, the statistic (z) and the p-value (*p*) are presented.

| **Predictor** | **OR** | **SE** | **z** | ***p^c^*** |
| --- | --- | --- | --- | --- |
| Effort^a^ |  |  |  |  |
| low *vs.* medium | 0.5140 | 0.0745 | -4.5900 | *** |
| low *vs.* high | 0.4900 | 0.0870 | -4.0170 | *** |
| medium *vs.* high | 0.9530 | 0.2143 | -0.2140 | >0.05 |
| Habitat |  |  |  |  |
| grassland *vs.* shrubland | 0.4400 | 0.0537 | -6.7240 | *** |
| grassland *vs.* forest | 0.8050 | 0.1147 | -1.5230 | >0.05 |
| shrubland *vs.* forest | 1.8280 | 0.2745 | 4.0190 | *** |
| Tick density^b^ |  |  |  |  |
| d1 *vs.* d2 | 0.5940 | 0.1230 | -2.5110 | >0.05 |
| d1 *vs.* d3 | 0.8220 | 0.1650 | -0.9730 | >0.05 |
| d1 *vs.* d4 | 0.8380 | 0.1640 | -0.9060 | >0.05 |
| d1 *vs.* d5 | 1.0870 | 0.2120 | 0.4290 | >0.05 |
| d2 *vs.* d3 | 1.3830 | 0.2000 | 2.2450 | >0.05 |
| d2 *vs.* d4 | 1.4090 | 0.1920 | 2.5120 | >0.05 |
| d2 *vs.* d5 | 1.8290 | 0.2470 | 4.4690 | *** |
| d3 *vs.* d4 | 1.0190 | 0.1300 | 0.1460 | >0.05 |
| d3 *vs.* d5 | 1.3220 | 0.1660 | 2.2200 | >0.05 |
| d4 *vs.* d5 | 1.2980 | 0.1510 | 2.2360 | >0.05 |

^a^low: 4 counts/plot, medium: 8 counts/plot, high: 12 counts/plot;

^b^d1=1/m^2^, d2=2/m^2^, d3=3/m^2^, d4=4/m^2^, d5=5/m^2^; ^c^*p<.05, **p<.01, ***p<.001.

**Supplementary Table S10.** Probability of capture of marked ticks as a function of habitat and effort estimated (‘lsmeans’ function of the R package ‘emmeans’) from the best fitted model for the CO_2_ trap method for the stage 1 of the experiment as presented in Table 4. The probability values (Prob.; scale: 0-1), the standard error of their estimate (SE) and the lower (LCL) and upper (UCL) limits of the estimated interval with 95% confidence are shown for each level of the categorical predictors of the model.

| **Habitat** | **Effort^a^** | **Prob.** | **SE** | **LCL** | **UCL** |
| --- | --- | --- | --- | --- | --- |
| Grassland | low | 0.0547 | 0.0096 | 0.0386 | 0.0770 |
|  | medium | 0.0867 | 0.0134 | 0.0637 | 0.1170 |
|  | *All* | *0.0690* | *0.0096* | *0.0523* | *0.0904* |
| Shrubland | low | 0.0913 | 0.0137 | 0.0678 | 0.1220 |
|  | medium | 0.1414 | 0.0240 | 0.1005 | 0.1950 |
|  | *All* | *0.1139* | *0.0156* | *0.0868* | *0.1482* |
| Forest | low | 0.0700 | 0.0143 | 0.0467 | 0.1040 |
|  | medium | 0.1098 | 0.0165 | 0.0814 | 0.1470 |
|  | *All* | *0.0879* | *0.0136* | *0.0646* | *0.1186* |
| *All* | *low* | *0.0706* | *0.0090* | *0.0549* | *0.0902* |
|  | *medium* | *0.1107* | *0.0117* | *0.0898* | *0.1357* |

^a^low: 1 CO_2_ trap/plot, medium, 2 CO_2_ traps/plot.

**Supplementary Table S11.** Odds ratios resulting from the paired comparison of the different levels of the categorical predictors (effort, habitat, and tick density in the experimental plots) of the CO_2_ trap-specific model with best fit of stage 1 of the experiment (Table 4) estimated using the 'lsmeans' function of the 'emmeans' package of R. The odds ratio (OR) value of each paired comparison, the standard error (SE) of that value, the statistic (z) and the p-value (*p*) are presented.

| **Predictor** | **OR** | **SE** | **z** | ***p^c^*** |
| --- | --- | --- | --- | --- |
| Effort^a^ |  |  |  |  |
| low *vs.* medium | 0.6100 | 0.1180 | -2.5580 | * |
| Habitat |  |  |  |  |
| grassland *vs.* shrubland | 0.5760 | 0.1200 | -2.6490 | * |
| grassland *vs.* forest | 0.7690 | 0.1710 | -1.1770 | >0.05 |
| shrubland *vs.* forest | 1.3350 | 0.3520 | 1.0960 | >0.05 |
| Tick density^b^ |  |  |  |  |
| d1 *vs.* d2 | 1.0000 | 0.3100 | 0.0000 | >0.05 |
| d1 *vs.* d3 | 0.8850 | 0.2560 | -0.4230 | >0.05 |
| d1 *vs.* d4 | 1.0330 | 0.2930 | 0.1140 | >0.05 |
| d1 *vs.* d5 | 2.5800 | 0.7820 | 3.1260 | * |
| d2 *vs.* d3 | 0.8850 | 0.2010 | -0.5390 | >0.05 |
| d2 *vs.* d4 | 1.0330 | 0.2270 | 0.1470 | >0.05 |
| d2 *vs.* d5 | 2.5800 | 0.6320 | 3.8710 | ** |
| d3 *vs.* d4 | 1.1670 | 0.2210 | 0.8160 | >0.05 |
| d3 *vs.* d5 | 2.9150 | 0.6350 | 4.9110 | *** |
| d4 *vs.* d5 | 2.4980 | 0.5260 | 4.3470 | *** |

^a^low: 1 CO_2_ trap/plot, medium: 2 CO_2_ traps/plot; ^b^d1=1/m^2^, d2=2/m^2^, d3=3/m^2^, d4=4/m^2^, d5=5/m^2^; ^c^*p<.05, **p<.01, ***p<.001.

**Supplementary Table S12**. Summary of the records of wild ungulates obtained from the camera traps placed in the study plots both in stages 1 (Exp.1) and 2 (Exp.2) of the experiment. The table shows both the number of visits recorded and the total number of images taken per wild ungulate species throughout the habitat of the study plots.

| **Experiment stage** | **Habitat** | **Red deer** | | **Roe deer** | | **Wild boar** | |
| --- | --- | --- | --- | --- | --- | --- | --- |
|  |  | **No. visits** | **No. images** | **No. visits** | **No. images** | **No. visits** | **No. images** |
| Exp.1 | Grassland | 101 | 2611 | 2 | 12 | 2 | 4 |
|  | Shrubland | 185 | 2705 | 3 | 48 | 22 | 40 |
|  | Forest | 38 | 152 | 11 | 44 | 15 | 135 |
| **Subtotal Exp.1** | | **324** | **5468** | **16** | **104** | **40** | **179** |
| Exp.2 | Grassland | 85 | 664 | 0 | 0 | 0 | 0 |
|  | Shrubland | 12 | 303 | 0 | 0 | 0 | 0 |
|  | Forest | 43 | 342 | 1 | 4 | 2 | 6 |
| **Subtotal Exp.1** | | **141** | **1309** | **1** | **4** | **2** | **6** |

**Supplementary Table S13.** Average cumulative capture efficacy (%) on day three of consecutive three-day surveys performed in stage 2 of the experiment. Results are shown per capture method and effort throughout habitat type and tick density. Efforts were low (1m drags/flags, four ASC, and 1 CO_2_ trap per plot), medium (5m drags/flags and eight ASC), and high (10m drags/flags and twelve ASC).

|  |  | **BD^a^** | | | **BF^b^** | | | | **ASC^c^** | | | | | **CDT^d^** | |  |
| --- | --- | --- | --- | --- | --- | --- | --- | --- | --- | --- | --- | --- | --- | --- | --- | --- |
|  | Ticks/m^2^ | 1m | 5m | 10m | 1m | 5m | 10m | | 4 | | 8 | 12 | | 1 | |  |
| Grassland | 2 | 0.0 | 5.6 | 0.0 | 0.0 | 0.0 | | 0.0 | 55.6 | 77.8 | | | 22.2 | | 27.8 | |
|  | 3 | 0.0 | 3.7 | 0.0 | 0.0 | 0.0 | | 3.7 | 40.7 | 44.4 | | | 7.4 | | 25.9 | |
|  | 4 | 2.8 | 5.6 | 0.0 | 2.8 | 0.0 | | 5.6 | 58.3 | 30.6 | | | 69.4 | | 33.3 | |
|  | *Average* | *0.9* | *4.9* | *0.0* | *0.9* | *0.0* | | *3.1* | *51.5* | *50.9* | | | *33.0* | | *29.0* | |
| Shrubland | 2 | 0.0 | 0.0 | 0.0 | 0.0 | 0.0 | | 5.6 | 77.8 | 55.6 | | | 55.6 | | 5.6 | |
|  | 3 | 3.7 | 0.0 | 0.0 | 3.7 | 3.7 | | 7.4 | 55.6 | 70.4 | | | 25.9 | | 18.5 | |
|  | 4 | 0.0 | 2.8 | 5.6 | 0.0 | 5.6 | | 8.3 | 83.3 | 83.3 | | | 72.2 | | 2.8 | |
|  | *Average* | *1.2* | *0.9* | *1.9* | *1.2* | *3.1* | | *7.1* | *72.2* | *69.8* | | | *51.2* | | *9.0* | |
| Forest | 2 | 0.0 | 5.6 | 0.0 | 0.0 | 0.0 | | 0.0 | 83.3 | 44.4 | | | 44.4 | | 27.8 | |
|  | 3 | 0.0 | 0.0 | 3.7 | 0.0 | 0.0 | | 0.0 | 74.1 | 51.9 | | | 25.9 | | 37.0 | |
|  | 4 | 2.8 | 0.0 | 2.8 | 2.8 | 0.0 | | 0.0 | 66.7 | 38.9 | | | 38.9 | | 16.7 | |
|  | *Average* | *0.9* | *1.9* | *2.2* | *0.9* | *0.0* | | *0.0* | *74.7* | *45.1* | | | *36.4* | | *27.2* | |
| Total | | 1.0 | 2.6 | 1.3 | 1.0 | 1.0 | | 3.4 | 66.2 | 55.2 | | | 40.2 | | 21.7 | |

**^a^**Blanket dragging; **^b^**Blanket flagging; ^c^Absolute surface count; ^d^CO_2_ traps.

**Supplementary Table S14.** Probability of accumulated capture of marked ticks at day three of consecutive three-day surveys as a function of tick density in the experimental plots and capture method estimated (‘lsmeans’ function of the R package ‘emmeans’) from the best fitted general model for the stage 2 of the experiment as presented in Table 6. The probability values (Prob.; scale: 0-1), the standard error of their estimate (SE) and the lower (LCL) and upper (UCL) limits of the estimated interval with 95% confidence are shown for each level of the categorical predictors of the model.

| **Tick density^a^** | **Method^b^** | **Prob.** | **SE** | **LCL** | **UCL** |
| --- | --- | --- | --- | --- | --- |
| d2 | BD | 0.0178 | 0.0053 | 0.0099 | 0.0319 |
|  | BF | 0.0215 | 0.0061 | 0.0123 | 0.0372 |
|  | ASC | 0.5431 | 0.0350 | 0.4741 | 0.6105 |
|  | CDT | 0.2023 | 0.0332 | 0.1449 | 0.2752 |
|  | *All* | *0.0948* | *0.0137* | *0.0712* | *0.1252* |
| d3 | BD | 0.0134 | 0.0040 | 0.0075 | 0.0238 |
|  | BF | 0.0161 | 0.0045 | 0.0094 | 0.0277 |
|  | ASC | 0.4706 | 0.0295 | 0.4134 | 0.5286 |
|  | CDT | 0.1595 | 0.0261 | 0.1146 | 0.2175 |
|  | *All* | *0.0726* | *0.0098* | *0.0557* | *0.0942* |
| d4 | BD | 0.0207 | 0.0058 | 0.0119 | 0.0358 |
|  | BF | 0.0249 | 0.0066 | 0.0149 | 0.0415 |
|  | ASC | 0.5807 | 0.0255 | 0.5300 | 0.6297 |
|  | CDT | 0.2281 | 0.0318 | 0.1719 | 0.2962 |
|  | *All* | *0.1087* | *0.0121* | *0.0872* | *0.1347* |
| *All* | *BD* | *0.0171* | *0.0047* | *0.0099* | *0.0292* |
|  | *BF* | *0.0205* | *0.0053* | *0.0124* | *0.0339* |
|  | *ASC* | *0.5317* | *0.0195* | *0.4934* | *0.5696* |
|  | *CDT* | *0.1950* | *0.0266* | *0.1480* | *0.2525* |

^a^d2=2/m^2^, d3=3/m^2^, d4=4/m^2^; ^b^BD: blanket dragging, BF: blanket flagging, ASC: absolute surface count, CDT: CO_2_ traps.

**Supplementary Table S15.** Odds ratios resulting from the paired comparison of the different levels of the categorical predictors (effort, habitat, and tick density in the experimental plots) of the model with best overall fit of stage 2 of the experiment (Table 6) estimated using the 'lsmeans' function of the 'emmeans' package of R. The odds ratio (OR) value of each paired comparison, the standard error (SE) of that value, the statistic (z) and the p-value (*p*) are presented.

| **Predictor** | **OR** | **SE** | **z** | ***p^c^*** |
| --- | --- | --- | --- | --- |
| Method^a^ |  |  |  |  |
| BD *vs.* BF | 0.8277 | 0.3176 | -0.4930 | >0.05 |
| BD *vs.* ASC | 0.0153 | 0.0044 | -14.3940 | *** |
| BD *vs.* CDT | 0.0716 | 0.0234 | -8.0820 | *** |
| BF *vs.* ASC | 0.0185 | 0.0051 | -14.5490 | *** |
| BF *vs.* CDT | 0.0865 | 0.0273 | -7.7490 | *** |
| ASC *vs.* CDT | 4.6856 | 0.8447 | 8.5680 | *** |
| Tick density^b^ |  |  |  |  |
| d2 *vs.* d3 | 1.3370 | 0.2349 | 1.6540 | >0.05 |
| d2 *vs.* d4 | 0.8580 | 0.1412 | -0.9280 | >0.05 |
| d3 *vs.* d4 | 0.6420 | 0.0946 | -3.0070 | ** |

^a^d2=2/m^2^, d3=3/m^2^, d4=4/m^2^; ^b^BD: blanket dragging, BF: blanket flagging, ASC: absolute surface count, CDT: CO_2_ traps; ^c^**p<.01, ***p<.001.

**Supplementary Table S16.** Probability of accumulated capture of marked ticks at day three of consecutive three-day surveys as a function of habitat and effort estimated (‘lsmeans’ function of the R package ‘emmeans’) from the best fitted model for the blanket dragging method for the stage 2 of the experiment as presented in Table 6. The probability values (Prob.; scale: 0-1), the standard error of their estimate (SE) and the lower (LCL) and upper (UCL) limits of the estimated interval with 95% confidence are shown for each level of the categorical predictors of the model.

| **Habitat** | **Effort^a^** | **Prob.** | **SE** | **LCL** | **UCL** |
| --- | --- | --- | --- | --- | --- |
| Grassland | low | 0.0005 | 0.0016 | 0.0000 | 0.1925 |
|  | medium | 0.0070 | 0.0089 | 0.0006 | 0.0802 |
|  | high | 0.0002 | 0.0009 | 0.0000 | 0.6135 |
|  | *All* | *0.0009* | *0.0027* | *0.0000* | *0.2210* |
| Shrubland | low | 0.0156 | 0.0139 | 0.0027 | 0.0858 |
|  | medium | 0.1763 | 0.3695 | 0.0015 | 0.9691 |
|  | high | 0.0062 | 0.0072 | 0.0006 | 0.0594 |
|  | *All* | *0.0268* | *0.0222* | *0.0052* | *0.1280* |
| Forest | low | 0.0346 | 0.0470 | 0.0023 | 0.3610 |
|  | medium | 0.3264 | 0.6966 | 0.0010 | 0.9959 |
|  | high | 0.0138 | 0.0094 | 0.0036 | 0.0514 |
|  | *All* | *0.0588* | *0.0780* | *0.0039* | *0.4970* |
| *All* | *low* | *0.0066* | *0.0053* | *0.0014* | *0.0310* |
|  | *medium* | *0.0827* | *0.1201* | *0.0040* | *0.6670* |
|  | *high* | *0.0026* | *0.0052* | *0.0001* | *0.1180* |

^a^low: 1 m drag/plot, medium: 5 m drag/plot, high: 10 m drag/plot.

**Supplementary Table S17.** Odds ratios resulting from the paired comparison of the different levels of the categorical predictors (effort, habitat, and tick density in the experimental plots) of the blanket dragging-specific model with best fit of stage 2 of the experiment (Table 6) estimated using the 'lsmeans' function of the 'emmeans' package of R. The odds ratio (OR) value of each paired comparison, the standard error (SE) of that value, the statistic (z) and the p-value (*p*) are presented.

| **Predictor** | **OR** | **SE** | **z** | ***p*** |
| --- | --- | --- | --- | --- |
| Effort^a^ |  |  |  |  |
| low *vs.* medium | 0.0740 | 0.1580 | -1.2210 | >0.05 |
| low *vs.* high | 2.5600 | 4.1370 | 0.5580 | >0.05 |
| medium *vs.* high | 34.6130 | 120.8840 | 1.0150 | >0.05 |
| Habitat |  |  |  |  |
| grassland *vs.* shrubland | 0.0330 | 0.1190 | -0.9460 | >0.05 |
| grassland *vs.* forest | 0.0146 | 0.0619 | -0.9970 | >0.05 |
| shrubland *vs.* forest | 0.4417 | 0.4284 | -0.8420 | >0.05 |
| Tick density^b^ |  |  |  |  |
| d2 *vs.* d3 | 1.0000 | 0.9210 | 0.0000 | >0.05 |
| d2 *vs.* d4 | 0.4910 | 0.3920 | -0.8900 | >0.05 |
| d3 *vs.* d4 | 0.4910 | 0.3360 | -1.0380 | >0.05 |

^a^low: 1 m drag/plot, medium: 5 m drag/plot, high: 10 m drag/plot; ^b^d2=2/m^2^, d3=3/m^2^, d4=4/m^2^.

**Supplementary Table S18.** Probability of accumulated capture of marked ticks at day three of consecutive three-day surveys as a function of habitat and effort estimated (‘lsmeans’ function of the R package ‘emmeans’) from the best fitted model for the blanket flagging method for the stage 2 of the experiment as presented in Table 6. The probability values (Prob.; scale: 0-1), the standard error of their estimate (SE) and the lower (LCL) and upper (UCL) limits of the estimated interval with 95% confidence are shown for each level of the categorical predictors of the model.

| **Habitat** | **Effort^a^** | **Prob.** | **SE** | **LCL** | **UCL** |
| --- | --- | --- | --- | --- | --- |
| Grassland | low | 0.0081 | 0.0109 | 0.0006 | 0.1044 |
|  | medium | 0.0022 | 0.0049 | 0.0000 | 0.1370 |
|  | high | 0.0271 | 0.0402 | 0.0014 | 0.3560 |
|  | *All* | *0.0079* | *0.0121* | *0.0004* | *0.1389* |
| Shrubland | low | 0.0312 | 0.0344 | 0.0034 | 0.2310 |
|  | medium | 0.0088 | 0.0095 | 0.0010 | 0.0697 |
|  | high | 0.0990 | 0.0808 | 0.0183 | 0.3931 |
|  | *All* | *0.0305* | *0.0208* | *0.0079* | *0.1107* |
| Forest | low | 0.0018 | 0.0022 | 0.0002 | 0.0193 |
|  | medium | 0.0005 | 0.0009 | 0.0000 | 0.0161 |
|  | high | 0.0059 | 0.0064 | 0.0007 | 0.0471 |
|  | *All* | *0.0017* | *0.0020* | *0.0002* | *0.0166* |
| *All* | *low* | *0.0077* | *0.0055* | *0.0019* | *0.0309* |
|  | *medium* | *0.0021* | *0.0030* | *0.0001* | *0.0332* |
|  | *high* | *0.0266* | *0.0156* | *0.0075* | *0.0839* |

^a^low: 1 m flag/plot, medium: 5 m flag/plot, high: 10 m flag/plot.

**Supplementary Table S19.** Odds ratios resulting from the paired comparison of the different levels of the categorical predictors (effort, habitat, and tick density in the experimental plots) of the blanket flagging-specific model with best fit of stage 2 of the experiment (Table 6) estimated using the 'lsmeans' function of the 'emmeans' package of R. The odds ratio (OR) value of each paired comparison, the standard error (SE) of that value, the statistic (z) and the p-value (*p*) are presented.

| **Predictor** | **OR** | **SE** | **z** | ***p^c^*** |
| --- | --- | --- | --- | --- |
| Effort^a^ |  |  |  |  |
| low *vs.* medium | 3.6454 | 5.7270 | 0.8230 | >0.05 |
| low *vs.* high | 0.2930 | 0.2290 | -1.5710 | >0.05 |
| medium *vs.* high | 0.0804 | 0.1250 | -1.6200 | >0.05 |
| Habitat |  |  |  |  |
| grassland *vs.* shrubland | 0.2540 | 0.5160 | -0.6750 | >0.05 |
| grassland *vs.* forest | 4.6690 | 8.8780 | 0.8100 | >0.05 |
| shrubland *vs.* forest | 18.4140 | 22.8000 | 2.3530 | * |
| Tick density^b^ |  |  |  |  |
| d2 *vs.* d3 | 0.2910 | 0.3220 | -1.1160 | >0.05 |
| d2 *vs.* d4 | 0.2120 | 0.2260 | -1.4570 | >0.05 |
| d3 *vs.* d4 | 0.7300 | 0.4170 | -0.5520 | >0.05 |

^a^low: 1 m flag/plot, medium: 5 m flag/plot, high: 10 m flag/plot; ^b^d2=2/m^2^, d3=3/m^2^, d4=4/m^2^; ^c^*p<.05.

**Supplementary Table S20.** Probability of accumulated capture of marked ticks at day three of consecutive three-day surveys as a function of habitat and effort estimated (‘lsmeans’ function of the R package ‘emmeans’) from the best fitted model for the absolute surface count method for the stage 2 of the experiment as presented in Table 6. The probability values (Prob.; scale: 0-1), the standard error of their estimate (SE) and the lower (LCL) and upper (UCL) limits of the estimated interval with 95% confidence are shown for each level of the categorical predictors of the model.

| **Habitat** | **Effort^a^** | **Prob.** | **SE** | **LCL** | **UCL** |
| --- | --- | --- | --- | --- | --- |
| Grassland | low | 0.6260 | 0.0655 | 0.4920 | 0.7430 |
|  | medium | 0.4820 | 0.0506 | 0.3840 | 0.5800 |
|  | high | 0.3960 | 0.0762 | 0.2600 | 0.5510 |
|  | *All* | *0.5020* | *0.0571* | *0.3920* | *0.6120* |
| Shrubland | low | 0.7580 | 0.0361 | 0.6810 | 0.8220 |
|  | medium | 0.6360 | 0.0563 | 0.5200 | 0.7370 |
|  | high | 0.5520 | 0.0451 | 0.4630 | 0.6380 |
|  | *All* | *0.6540* | *0.0337* | *0.5850* | *0.7170* |
| Forest | low | 0.5920 | 0.0515 | 0.4890 | 0.6880 |
|  | medium | 0.4470 | 0.0768 | 0.3050 | 0.5970 |
|  | high | 0.3630 | 0.0431 | 0.2840 | 0.4510 |
|  | *All* | *0.4670* | *0.0337* | *0.3740* | *0.5610* |
| *All* | *low* | *0.6630* | *0.0342* | *0.5930* | *0.7270* |
|  | *medium* | *0.5220* | *0.0466* | *0.4310* | *0.6120* |
|  | *high* | *0.4360* | *0.0395* | *0.3610* | *0.5140* |

^a^low: 4 counts/plot, medium: 8 counts/plot, high: 12 counts/plot.

**Supplementary Table S21.** Odds ratios resulting from the paired comparison of the different levels of the categorical predictors (effort, habitat, and tick density in the experimental plots) of the absolute surface count-specific model with best fit of stage 2 of the experiment (Table 6) estimated using the 'lsmeans' function of the 'emmeans' package of R. The odds ratio (OR) value of each paired comparison, the standard error (SE) of that value, the statistic (z) and the p-value (*p*) are presented.

| **Predictor** | **OR** | **SE** | **z** | ***p^c^*** |
| --- | --- | --- | --- | --- |
| Effort^a^ |  |  |  |  |
| low *vs.* medium | 1.8000 | 0.4810 | 2.2010 | >0.05 |
| low *vs.* high | 2.5500 | 0.5140 | 4.6370 | *** |
| medium *vs.* high | 1.4200 | 0.4030 | 1.2180 | >0.05 |
| Habitat |  |  |  |  |
| grassland *vs.* shrubland | 0.5330 | 0.1600 | -2.0990 | >0.05 |
| grassland *vs.* forest | 1.1510 | 0.4300 | 0.3780 | >0.05 |
| shrubland *vs.* forest | 2.1610 | 0.4660 | 3.5760 | ** |
| Tick density^b^ |  |  |  |  |
| d2 *vs.* d3 | 1.7890 | 0.3814 | 2.7300 | * |
| d2 *vs.* d4 | 0.8840 | 0.1792 | -0.6100 | >0.05 |
| d3 *vs.* d4 | 0.4940 | 0.0885 | -3.9360 | *** |

^a^low: 4 counts/plot, medium: 8 counts/plot, high: 12 counts/plot; ^b^d2=2/m^2^, d3=3/m^2^, d4=4/m^2^; ^c^*p<.05, **p<.01, ***p<.001.

**Supplementary Table S22.** Probability of accumulated capture of marked ticks at day three of consecutive three-day surveys as a function of habitat estimated (‘lsmeans’ function of the R package ‘emmeans’) from the best fitted model for the CO_2_ trap method for the stage 2 of the experiment as presented in Table 6. The probability values (Prob.; scale: 0-1), the standard error of their estimate (SE) and the lower (LCL) and upper (UCL) limits of the estimated interval with 95% confidence are shown for each level of the categorical predictors of the model.

| **Habitat** | **Prob.** | **SE** | **LCL** | **UCL** |
| --- | --- | --- | --- | --- |
| Grassland | 0.2979 | 0.0521 | 0.2066 | 0.4090 |
| Shrubland | 0.0859 | 0.0314 | 0.0411 | 0.1710 |
| Forest | 0.2603 | 0.0499 | 0.1747 | 0.3690 |

**Supplementary Table S23.** Odds ratios resulting from the paired comparison of the different levels of the categorical predictors (habitat and tick density in the experimental plots) of the CO_2_ trap-specific model with best fit of stage 2 of the experiment (Table 6) estimated using the 'lsmeans' function of the 'emmeans' package of R. The odds ratio (OR) value of each paired comparison, the standard error (SE) of that value, the statistic (z) and the p-value (*p*) are presented.

| **Predictor** | **OR** | **SE** | **z** | ***P^b^*** |
| --- | --- | --- | --- | --- |
| Habitat |  |  |  |  |
| grassland *vs.* shrubland | 4.5180 | 2.1080 | 3.2320 | ** |
| grassland *vs.* forest | 1.2060 | 0.4270 | 0.5300 | >0.05 |
| shrubland *vs.* forest | 0.2670 | 0.1260 | -2.7990 | * |
| Tick density^a^ |  |  |  |  |
| d2 *vs.* d3 | 0.6710 | 0.2900 | -0.9230 | >0.05 |
| d2 *vs.* d4 | 1.2090 | 0.5220 | 0.4380 | >0.05 |
| d3 *vs.* d4 | 1.8010 | 0.6580 | 1.6100 | >0.05 |

^a^d2=2/m^2^, d3=3/m^2^, d4=4/m^2^; ^b^*p<.05, **p<.01.

**Supplementary Figure S1.** Average accumulated time (minutes) of plot use by *Hyalomma lusitanicum* hosts as estimated from two camera traps per plot and habitat type in stage 1 of the experiment.


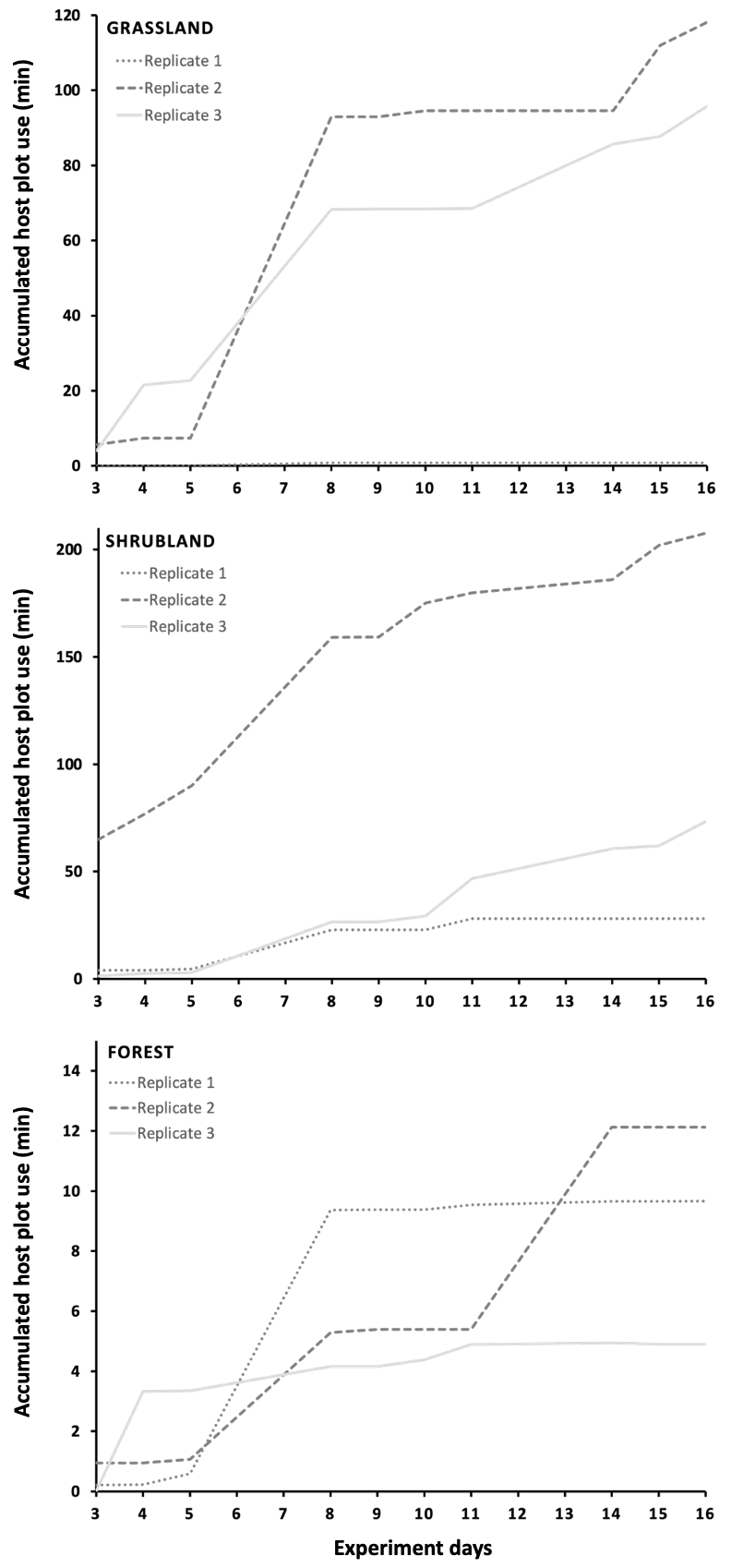


**Supplementary Figure S2.** Average accumulated time (minutes) of plot use by *Hyalomma lusitanicum* hosts as estimated from two camera traps per plot and habitat type in stage 2 of the experiment.


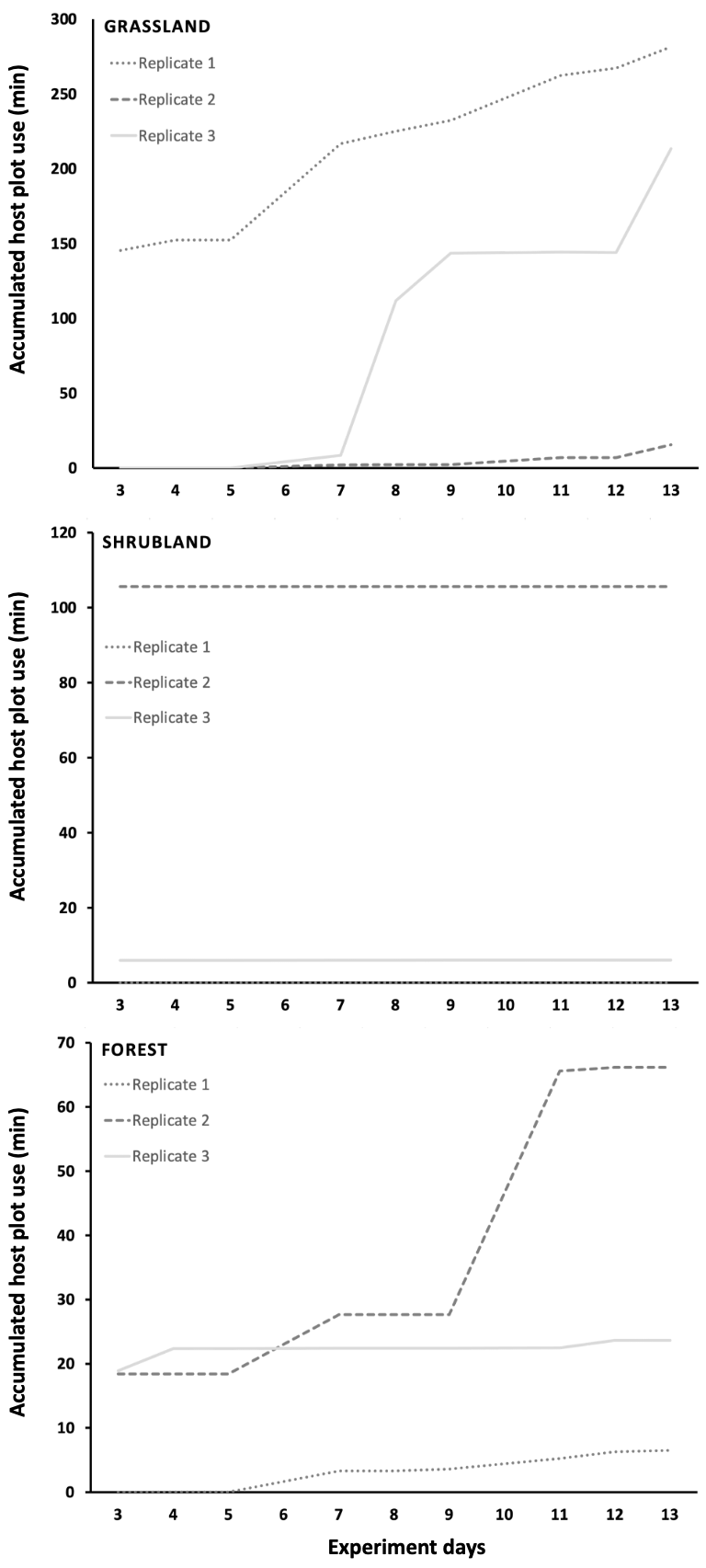


**Supplementary Figure S3.** Variation in average daily soil temperature (ºC; red line) and relative humidity (%; blue line) along experimental stages 1 and 2 as estimated from the registers of data loggers.


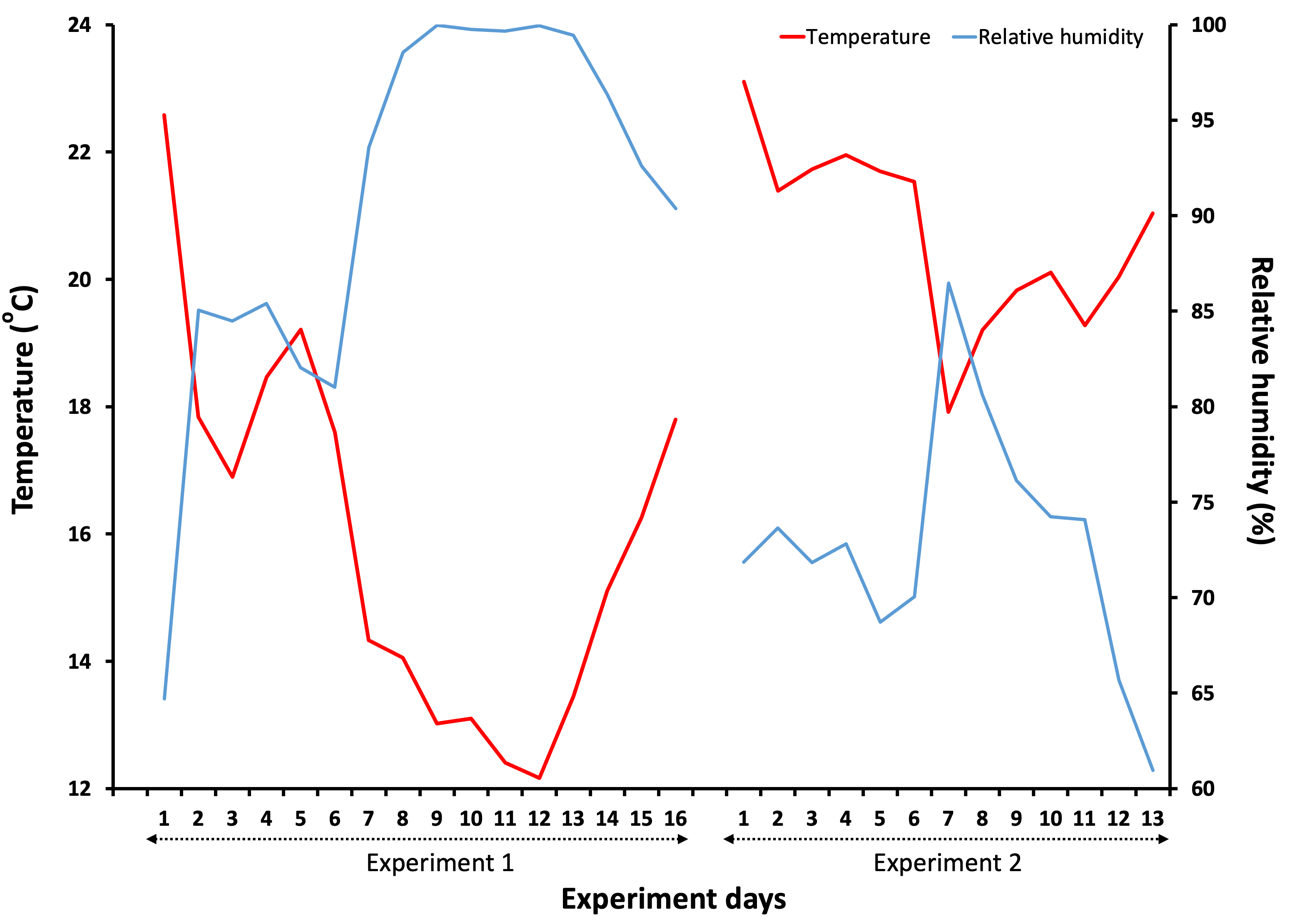


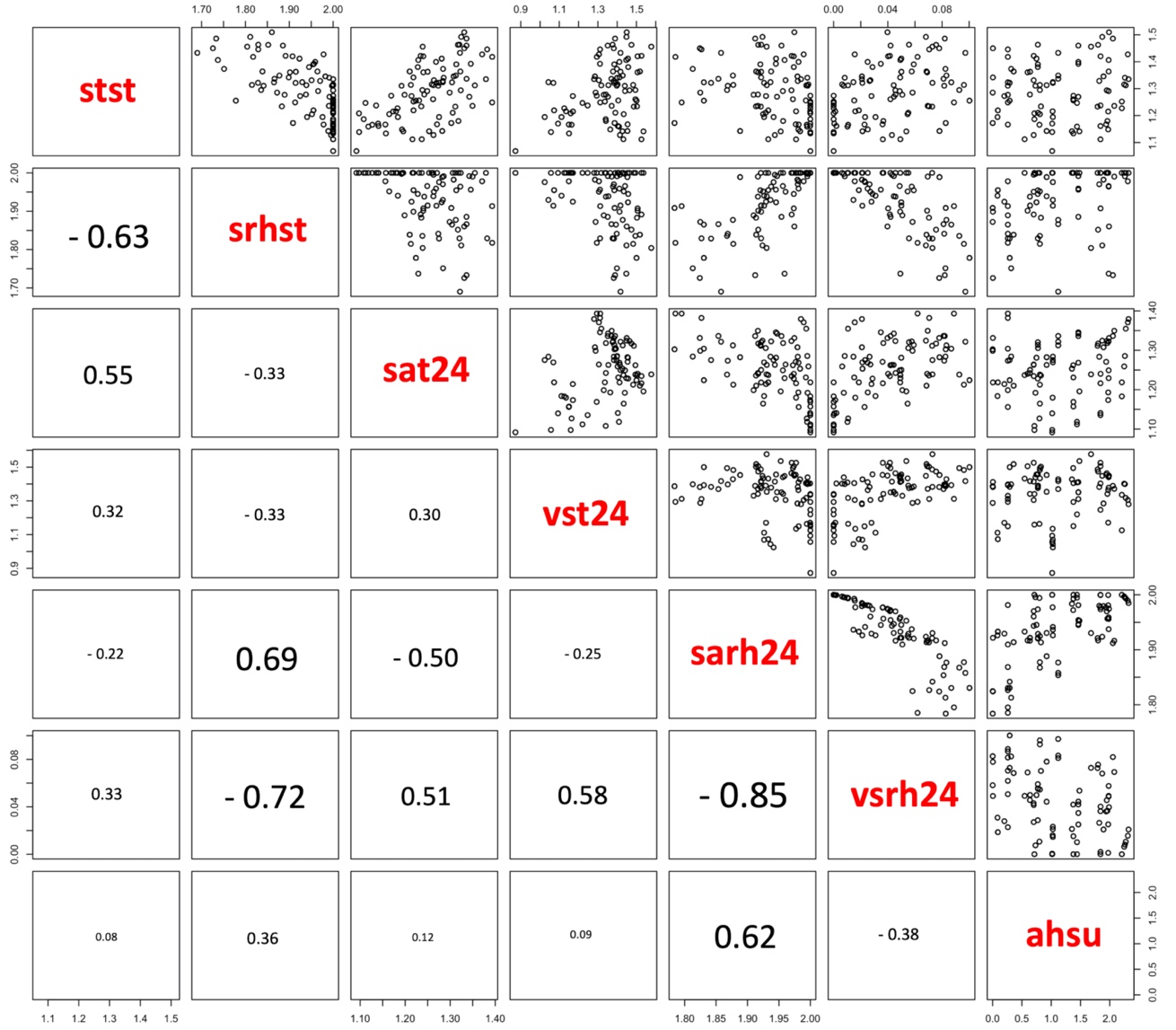
**Supplementary Figure S4.** Pearson correlation matrix between the continuous predictors estimated from camera trapping and data logger registers of soil temperature and relative humidity in stage 1 of the experiment. The Pearson correlation coefficient (r) of each paired relationship between predictors is shown in the lower left half of the chart.

**stst**: soil temperature (ºC) at sampling time, **srhst:** soil relative humidity (%) at sampling time, **sat24**: soil average temperature (ºC) in the 24h before sampling, **vst24**: variation in soil temperature registers (ºC) in the previous 24h to sampling, **sarh24**: soil average relative humidity (%) in the previous 24h to sampling, **vsrh24**: variation in soil relative humidity registers (%) in the 24h before sampling, **ahsu**: accumulated host (wild ungulates) space use of the experimental plots.


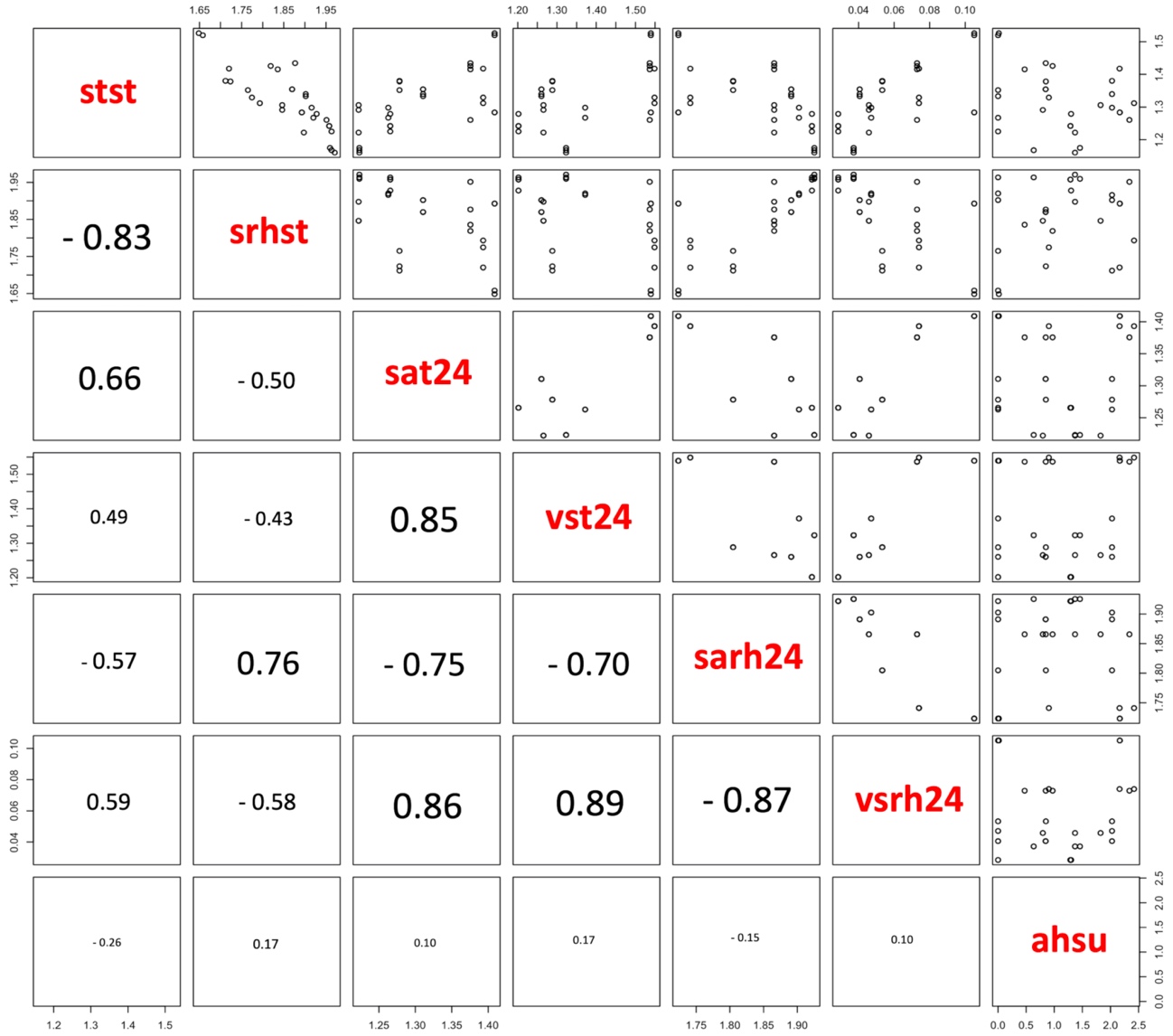
**Supplementary Figure S5.** Pearson correlation matrix between the continuous predictors estimated from camera trapping and data logger registers of soil temperature and relative humidity in stage 2 of the experiment. The Pearson correlation coefficient (r) of each paired relationship between predictors is shown in the lower left half of the chart.

**stst**: soil temperature (ºC) at sampling time, **srhst:** soil relative humidity (%) at sampling time, **sat24**: soil average temperature (ºC) in the 24h before sampling, **vst24**: variation in soil temperature registers (ºC) in the previous 24h to sampling, **sarh24**: soil average relative humidity (%) in the previous 24h to sampling, **vsrh24**: variation in soil relative humidity registers (%) in the 24h before sampling, **ahsu**: accumulated host (wild ungulates) space use of the experimental plots.
